# Highly Precise and Developmentally Programmed Genome Assembly in *Paramecium* Requires Ligase IV–Dependent End Joining

**DOI:** 10.1371/journal.pgen.1002049

**Published:** 2011-04-14

**Authors:** Aurélie Kapusta, Atsushi Matsuda, Antoine Marmignon, Michael Ku, Aude Silve, Eric Meyer, James D. Forney, Sophie Malinsky, Mireille Bétermier

**Affiliations:** 1 CNRS UPR3404, Centre de Génétique Moléculaire, Gif-sur-Yvette, France; 2 Université Paris 11, Département de Biologie, Orsay, France; 3 CNRS FRC3115, Centre de Recherches de Gif–sur-Yvette, Gif-sur-Yvette, France; 4 Department of Biochemistry, Purdue University, West Lafayette, Indiana, United States of America; 5 Institut de Biologie de l'Ecole Normale Supérieure, CNRS UMR8197, INSERM U1024, Paris, France; 6 Université Paris Diderot – Paris 7, UFR des Sciences du Vivant, Paris, France; The University of North Carolina at Chapel Hill, United States of America

## Abstract

During the sexual cycle of the ciliate *Paramecium*, assembly of the somatic genome includes the precise excision of tens of thousands of short, non-coding germline sequences (Internal Eliminated Sequences or IESs), each one flanked by two TA dinucleotides. It has been reported previously that these genome rearrangements are initiated by the introduction of developmentally programmed DNA double-strand breaks (DSBs), which depend on the domesticated transposase PiggyMac. These DSBs all exhibit a characteristic geometry, with 4-base 5′ overhangs centered on the conserved TA, and may readily align and undergo ligation with minimal processing. However, the molecular steps and actors involved in the final and precise assembly of somatic genes have remained unknown. We demonstrate here that Ligase IV and Xrcc4p, core components of the non-homologous end-joining pathway (NHEJ), are required both for the repair of IES excision sites and for the circularization of excised IESs. The transcription of *LIG4* and *XRCC4* is induced early during the sexual cycle and a Lig4p-GFP fusion protein accumulates in the developing somatic nucleus by the time IES excision takes place. RNAi–mediated silencing of either gene results in the persistence of free broken DNA ends, apparently protected against extensive resection. At the nucleotide level, controlled removal of the 5′-terminal nucleotide occurs normally in *LIG4*-silenced cells, while nucleotide addition to the 3′ ends of the breaks is blocked, together with the final joining step, indicative of a coupling between NHEJ polymerase and ligase activities. Taken together, our data indicate that IES excision is a “cut-and-close” mechanism, which involves the introduction of initiating double-strand cleavages at both ends of each IES, followed by DSB repair *via* highly precise end joining. This work broadens our current view on how the cellular NHEJ pathway has cooperated with domesticated transposases for the emergence of new mechanisms involved in genome dynamics.

## Author Summary

Double-strand breaks (DSBs) are among the most deleterious lesions that may occur on DNA. Some physiological processes, however, involve the introduction of DSBs and their subsequent repair. In the ciliate *Paramecium*, programmed DSBs initiate the extensive genome rearrangements that take place at each sexual cycle, during the development of the somatic nucleus. In particular, short intervening germline sequences (one every 1–2 kb along the genome) are spliced out from coding and non-coding regions. In this study, we present evidence that this process is a two-step mechanism and involves DNA cleavage at both ends of each excised sequence, followed by DSB repair. We demonstrate that cellular end-joining proteins, Ligase IV and its partner, Xrcc4p, are essential for the closure of broken excision sites, which has to be precise at the nucleotide level to allow the assembly of functional genes. This precision stands in sharp contrast to the notion that end joining is an error-prone DSB repair pathway. Therefore, *Paramecium* provides an excellent model for analysis of an intrinsically precise end joining pathway that has been recruited for genome-wide DSB repair.

## Introduction

Although double-strand breaks (DSBs) are among the most deleterious DNA lesions, essential cellular processes involve the programmed introduction of DSBs and their subsequent repair by various pathways. During meiosis, double-strand DNA cleavage mediated by the topoisomerase II-like Spo11 endonuclease triggers homologous recombination, which is essential for the correct segregation of homologs and the mixing of parental alleles without DNA loss (see [1] for review). During lymphocyte differentiation in vertebrates, V(D)J recombination drives the generation of immunoglobulin and T-cell receptor diversity (reviewed in [2]). Programmed DSBs are introduced at both ends of non-coding intervening sequences by the Rag1 domesticated transposase [3] and its partner, Rag2 (reviewed in [4]). Assembly of coding segments then relies on non-homologous end joining (NHEJ) (reviewed in [5]): in this DSB repair pathway, binding of the Ku70/Ku80 heterodimer to DNA ends facilitates their synapsis and recruits other factors involved in their processing and ligation. In the final step, Ligase IV and its partner Xrcc4p are required for covalent joining of the two broken ends.

In ciliates, massive genome rearrangements initiated by developmentally programmed DSBs are associated with DNA elimination during nuclear differentiation [6], [7]. In these unicellular eukaryotes, two kinds of nuclei coexist in the same cytoplasm [8]: the somatic macronucleus (MAC) is essential for gene expression but is destroyed at each sexual cycle, while the germline micronucleus (MIC) undergoes meiosis and transmits its genome to the zygotic nucleus. New MICs and MACs of sexual progeny differentiate from copies of the zygotic nucleus and extensive genome rearrangements take place in the new MAC during this process. In *Paramecium tetraurelia*, MAC development involves DNA amplification (from 2n to 800–1000n) and elimination of two types of germline sequences. Regions extending up to several kb, often including repeated DNA, are eliminated in an imprecise manner, leading to chromosome fragmentation or to internal deletions [9]. In addition, an estimated 60,000 single-copy, short and non coding Internal Eliminated Sequences (IES) are spliced out during assembly of functional genes (reviewed in [10]). *Paramecium* IESs are invariably flanked by two TA dinucleotides, one copy of which is left at their excision site on MAC chromosomes. Their excision is initiated by 4-bp staggered DSBs centered on these conserved TAs [11] and PiggyMac, a domesticated transposase from the *piggyBac* family, is essential for DNA cleavage [12]. Given the predicted density of IESs in the genome, excision would lead to around one DSB every 1–2 kb within the developing MAC.

The question addressed in this study is how *Paramecium* processes DSBs to achieve the precise assembly of somatic genes. Careful examination of cleaved IES ends has led to a mechanistic model, in which two DSBs, one at each end, initiate IES excision [11]. Genetic evidence for a crosstalk between ends before DNA cleavage further supported the view that IES ends are recognized or cleaved in a concerted manner [13]. It was proposed, therefore, that an end-joining DSB repair activity carries out the closure of excision sites on MAC chromosomes. However, alternative models involving a single initiating DSB (at either end) followed by DNA transesterification, have not been definitively ruled out. Indeed, molecules with a single cleaved end are detected in *P. tetraurelia* [13]: nucleophilic attack of the other IES boundary by the free flanking DNA could directly assemble a MAC junction and liberate a linear IES, as proposed for the related ciliate *Tetrahymena* [14]. Alternatively, attack from the free IES end would directly circularize the IES and leave a DSB at the excision site, as suggested for the more distant ciliate *Oxytricha* [15]. In *Paramecium*, linear and circular IES molecules are produced during MAC development [16], making it difficult to draw firm conclusions in favor of any model. Identifying the pathway(s) involved in the last steps of IES excision (assembly of chromosome and circle junctions) should provide new insight into how initial cleavages are introduced at IES ends.

We report here that an end-joining pathway is required for IES excision. Focusing on ATP-dependent DNA ligases, we identified nine genes in *P. tetraurelia*, two of which encode homologs of Ligase IV, an essential actor of the NHEJ pathway. We also found one *XRCC4* homolog. *LIG4* and *XRCC4* genes are specifically induced during sexual processes, before MAC development starts, and a Lig4p-GFP fusion localizes to the developing new MAC, in which IES excision takes place. Functional inactivation of *LIG4* or *XRCC4* completely abolishes the formation of chromosome and circle junctions, demonstrating that IES excision is initiated by two DSBs introduced at both ends of each IES, followed by Ligase IV/Xrcc4p-dependent repair. We propose that the remarkable precision in end joining is largely driven by the characteristic structure of the broken ends generated during IES excision.

## Results

### ATP-dependent DNA ligases in *P. tetraurelia*

**Figure 1:**
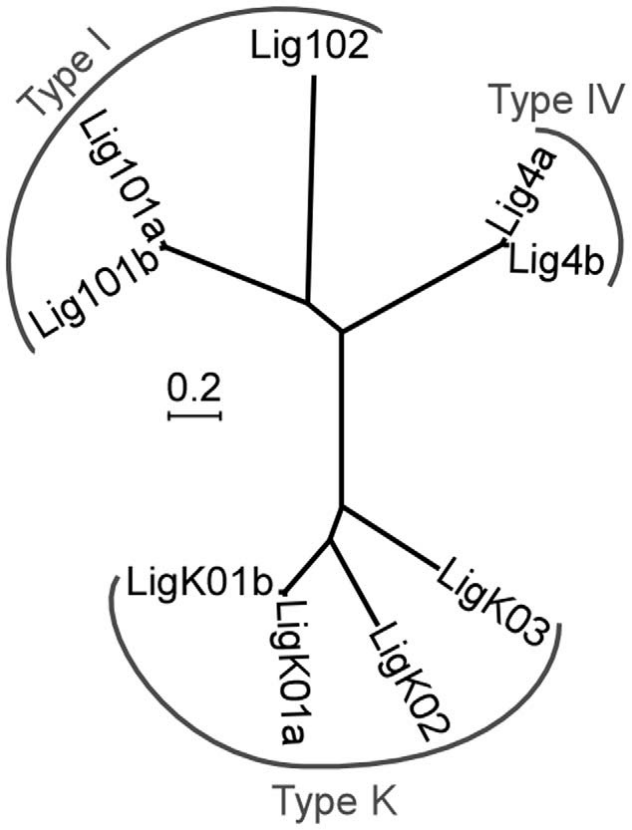
Neighbor-joining tree of *P. tetraurelia* ATP-dependent DNA ligases. Ligases produced from ohnologous genes are designated as “a” and “b”. The tree is based on protein sequences using the following parameters: bootstrap 1000, pairwise deletion of gaps, Poisson correction, uniform rates among sites. Accession numbers in ParameciumDB: *LIG101a* (*GSPATG00024948001*), *LIG101b* (*PTETG9500001001*), *LIG102* (*GSPATG00030449001*), *LIG4a* (*PTETG5400008001*), *LIG4b* (*PTETG7200002001*), *LIGK01a* (*GSPATG00034046001*), *LIGK01b* (*GSPATG00037262001*), *LIGK02* (*PTETG100004001*), *LIGK03* (*GSPATG00025612001*). The scale indicates the number of amino acid substitutions at each site between related proteins.

As a first step towards understanding the role of end joining pathways in IES excision, we searched for ATP-dependent DNA ligases encoded by the *P. tetraurelia* MAC genome and found nine putative genes, based on sequence homology. Some of them grouped in pairs with high nucleotide sequence identity (Figure 1), as a consequence of the most recent whole genome duplication (WGD) that occurred during evolution of the *P. aurelia* group of species [17]: these duplicated genes will be designated as ohnologs.

Three major families of ATP-dependent DNA ligases have been described in eukaryotes [18]. Type I ligases (Lig1p) are mainly involved in the ligation of discontinuous Okasaki fragments during replication and in the repair of DNA nicks. Type III ligases (Lig3p) are specialized in the repair of single-strand lesions in the nucleus and in mitochondria: they are restricted to metazoa and Lig1p can perform their function in other organisms. Finally, type IV ligases (Lig4p) are strictly essential for DSB repair *via* the NHEJ pathway. To classify *Paramecium* ligases, we constructed a phylogenetic tree with 52 other ATP-dependent DNA ligases from various organisms, including prokaryotes (Figure S1). We readily identified three *Paramecium* Lig1p and two Lig4p homologs but no clear Lig3p (Figure 1). The last four *P. tetraurelia* ligases formed a monophyletic group with type K ligases, diverged ligases first identified in kinetoplastid protozoan parasites, where they are involved in maintenance of mitochondrial kDNA [19], [20].

Alternative end-joining pathways have been reported and differ with regard to the precision of junction formation (reviewed in [21]). Microhomology-mediated end joining (MMEJ) involves end resection and may use Lig1p or type K ligases [22] to generate heterogeneous junctions. In contrast, the canonical NHEJ pathway is able to repair DSBs precisely, without any nucleotide loss, and relies on Lig4p. Because *Paramecium* IES excision is highly precise, we concentrated on this pathway and searched the *P. tetraurelia* genome for genes encoding Xrcc4p, the other essential component of the NHEJ ligation complex [23]. Thorough *in silico* analyses identified one *XRCC4* homolog, in spite of low primary sequence conservation (Figure S2). We also found two putative *XLF/Cernunnos* genes (Figure S3), which encode a cofactor of the Lig4p-Xrcc4p complex [24], [25]. We focused our work on *LIG4* and *XRCC4*, because null mutations in these two genes result in embryonic lethality in mouse [26]-[28].

### Developmentally programmed expression of *LIG4* and *XRCC4*

**Figure 2:**
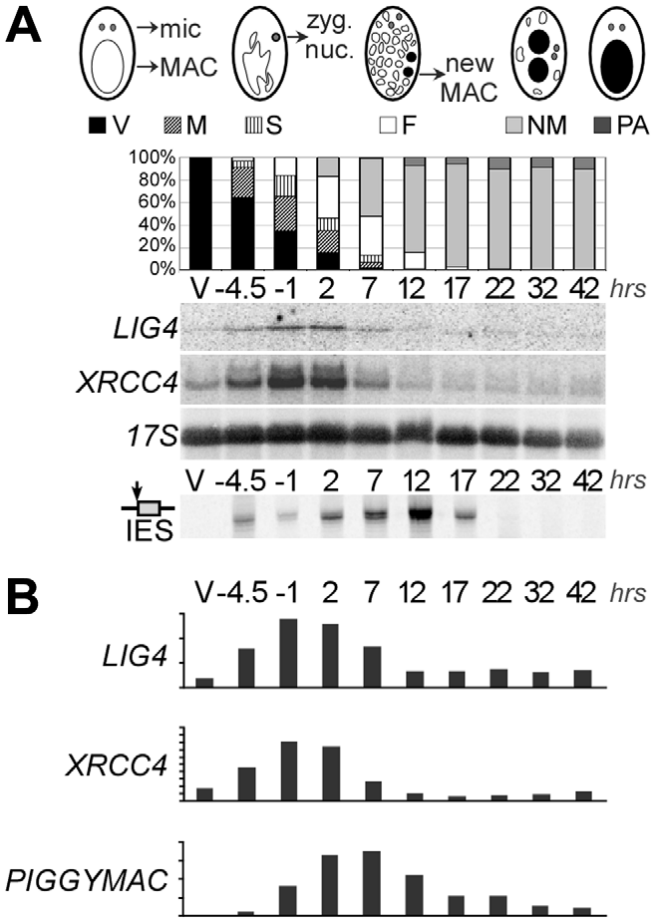
Induction of *LIG4* and *XRCC4* transcription during autogamy. A. Northern blot hybridization of total RNA extracted from strain 51 during an autogamy time-course in standard growth medium. The blot was hybridized successively with *LIG4*, *XRCC4* (see maps in Figure 4A) and 17S rDNA probes. The histograms show the progression of autogamy, with the different cellular stages diagrammed on top. Bottom panel shows the LMPCR-mediated detection of DSBs at the left boundary of IES sm19-576 (broken MAC ends). V: vegetative cells. The following time-points are indicated in hrs. Time 0 corresponds to 50% of the cells harboring a fragmented old MAC. B. Quantification of mRNA levels from the blots shown in A. For *PiggyMac,* values were calculated from a previous hybridization of the same blot [12]. 17S rRNA signal was used for normalization. Y-axes are in arbitrary units.

To monitor the transcription of *LIG4* genes, we hybridized northern blots with a *LIG4a* probe. This fragment shared 91% identity with the corresponding region in *LIG4b* and therefore revealed both *LIG4* ohnologs. We detected basal levels of mRNA of the expected size in vegetative cells (Figure 2A). During autogamy, a self-fertilization sexual process, transient accumulation of *LIG4* transcripts was clearly observed at early time-points. The same induction pattern was observed for *XRCC4* and accumulation of both transcripts preceded that of *PiggyMac* mRNA, which encodes the putative IES excisase (Figure 2B). Because northern blot hybridization would not distinguish between *LIG4a* and *LIG4b* transcripts, we performed RT-PCR experiments to distinguish the expression of both genes: only *LIG4a* mRNA was consistently detected by RT-PCR in all experiments and found to be induced early during autogamy, while *LIG4b* transcripts were detected at lower levels and exhibited more variable patterns of expression (not shown). This suggests that most of the signal detected on northern blots may be attributed to *LIG4a* transcription. Early induction of *LIG4a* was clearly confirmed by the statistical analysis of microarrays from four independent autogamy time-course experiments [29], while no significant variation was observed for *LIG4b* throughout early autogamy stages (Figure S3). However, oligonucleotide microarrays do not allow comparing the absolute expression levels of both ohnologs. Microarray analysis also revealed an early induction pattern for *XRCC4* and for one *Cernunnos* ohnolog (*CERa*; see Figure S3).

**Figure 3:**
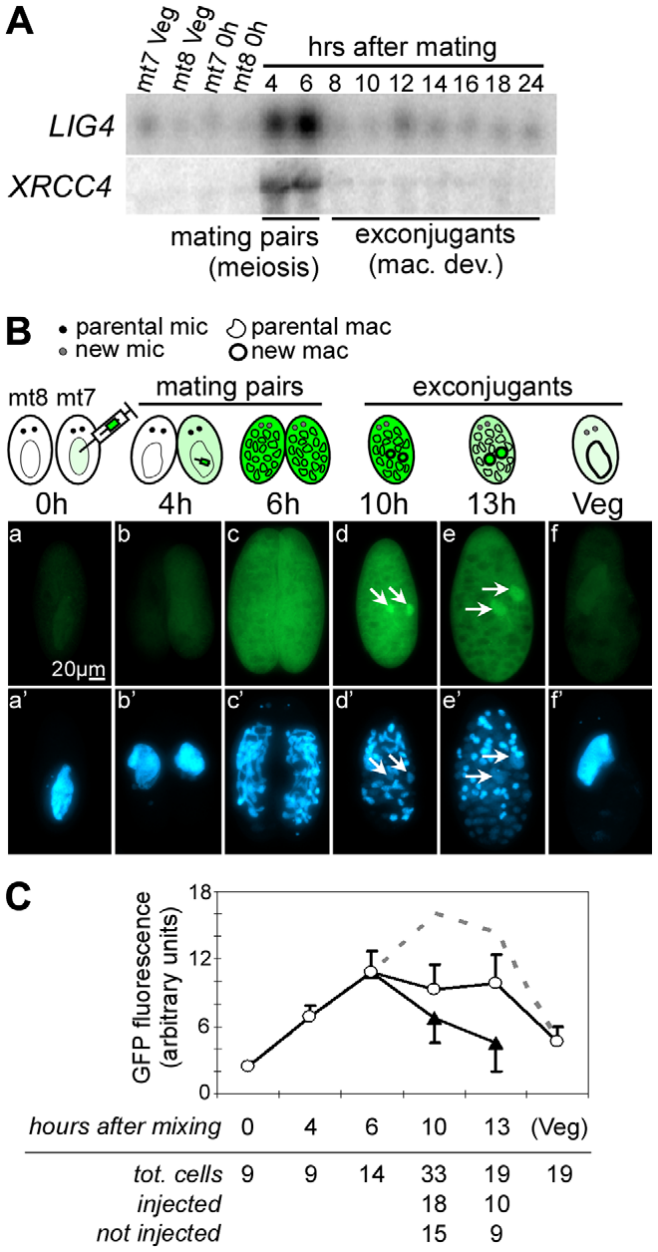
*LIG4* and *XRCC4* expression during synchronized conjugation. A. Northern blot hybridization of total RNA extracted during conjugation of d4-110 mt7 and mt8. *LIG4* and *XRCC4* probes (Figure 4A) were used successively. Mac. Dev.: MAC development. B. Localization of a Lig4a-GFP fusion during conjugation. Reactive mt7 cells transformed with plasmid pLIG401GC, and exhibiting a wild-type swimming behavior (see Materials and Methods), were mated with non injected d4-502 (mt8 *pwA*) at t = 0 hrs. At indicated time-points, cells were fixed and stained with DAPI before observation with a standard fluorescence microscope (GFP filter: a–f; DAPI filter: a’–f’). Developing new MACs are indicated with arrows. C. Quantification of intracellular GFP fluorescence. White circles represent the signal from strongly fluorescent cells originating from the transformed mt7 parent, black triangles represent exconjugants produced by the non-transformed mt8 partner, into which fluorescence has diffused during mating. The calculated total amount of Lig4a-GFP produced by a single transformant is represented as the sum of the two signals (dotted line). Bar  =  standard deviation.

Sexual reproduction in *Paramecium* includes MIC meiosis, followed by the development of new MACs. To identify more precisely the stage, at which *LIG4* and *XRCC4* are induced, we performed northern blot hybridizations of RNA samples extracted from conjugating cells. Indeed, during conjugation, MIC meiosis takes place within mating pairs and can be clearly distinguished from MAC development, which starts after pair separation [30]. Moreover, conjugation can be synchronized within a 1.5-hr time-window (against 5–6 hrs at best for autogamy), making it possible to separate the two events. For both *LIG4* and *XRCC4* genes, a peak of mRNA was clearly observed 4–6 hrs following mixing (Figure 3A), which corresponds to MIC meiosis, well before exconjugants separate (around 6–7 hrs) and new MACs start to differentiate. Therefore, induction of *LIG4* and *XRCC4* is not a response to DNA double-strand breaks introduced in the new MAC but is rather part of the developmental program of sexual processes.

### Lig4p accumulates in developing new MACs during IES excision

We used a *LIG4a-GFP* transgene expressed from the endogenous *LIG4a* promoter to follow the production and localization of the fluorescent fusion protein during synchronized conjugation. *P. tetraurelia* mt7 cells transformed with this transgene were mated with non-injected mt8 cells homozygous for the *pwA* mutation, which produces an abnormal swimming phenotype [31]: after pair separation, exconjugants issued from injected and non-injected cells were sorted according to their swimming behavior. GFP fluorescence was detected in the cytoplasm and MAC of starved injected cells (Figure 3B, panel a). Fluorescence increased during mating (panels b and c) and a strong signal was observed throughout the cells until 13 hrs and concentrated into developing new MACs at 10–13h rs (panels d and e), precisely at the time and place where IESs are massively excised [16]. It returned to background levels when cells resumed vegetative growth (panel f).

During mating, we observed that GFP fluorescence diffused from the injected cell to its non-injected partner (panel c). Therefore, to quantify the overall signal produced from the fusion transgene injected in the mt7 parent, we measured the total GFP fluorescence in vegetative injected cells, in mating pairs, and, separately, in exconjugants produced from injected (wild-type swimming behavior) and non-injected (mutant phenotype) cells. After the separation of exconjugants, the signal measured in non-injected cells (which rapidly lost their fluorescence following pair separation) was added to that of injected cells to calculate the total amount of protein produced by the transgene that was introduced initially in the mt7 parent (Figure 3C). This pointed to a peak of protein accumulation between 10 and 13 hrs following mating, concomitant with the concentration of the GFP fusion into the new MACs.

### RNAi against *LIG4* triggers regeneration of the old MAC during conjugation

**Figure 4:**
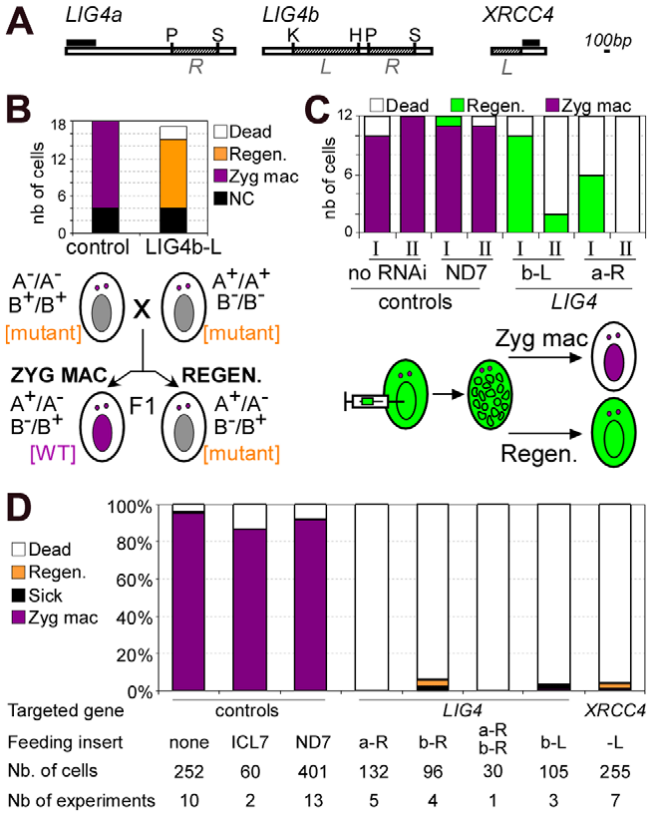
Analysis of sexual progeny of *LIG4*-silenced cells. A. Maps of *LIG4* and *XRCC4* genes. *XRCC4* accession number in ParameciumDB is *GSPATG00029540001*. The fragments used in the construction of RNAi plasmids are shown in hatched boxes and designated in italics (*L* or *R*). Probes used for Northern blot hybridization are displayed as black boxes: we used the first 510 bp of *LIG4a* open reading frame and a 302-bp fragment encompassing bp 508–809 for *XRCC4*. Restriction sites: P  =  *Pst*I, S  =  *Spe*I, K  =  *Kpn*I, H  =  *Hind*III. B. Survival and genetic analysis of F1 progeny from a cross between a3093 (mt7 *pwB*) and d4-502 (mt8 *pwA*). The histogram shows the number of cells surviving conjugation and the genotype of their MAC, out of 18 cells for the control experiment and 17 for the *LIG4* knock-down. NC: cells resulting from a conjugation failure within a mating pair (mutant phenotype in F1 and F2). Zyg. MAC: cells containing a functional zygotic nucleus. Regen: MAC regenerants in F1 (mutant phenotype in F1 and recovery of wild-type clones in post-autogamous F2). Control RNAi was carried out with an empty vector (no insert). The segregation of *pwA* (A-) and *pwB* (B-) markers in F1 MICs is shown below the histogram. The cell phenotype determined by the MAC is indicated between square brackets. C. MAC regeneration in post-autogamous progeny of 51 cells injected with a GFP transgene. Cells were transferred at day 0 in control (no RNAi or RNAi against *ND7*) or *LIG4*-silencing medium and, following starvation, 100% autogamy was observed at day 2. Survival tests were performed on 12-cell samples at day 2 (I) or day 4 (II). Regen: MAC regenerants, as judged by GFP fluorescence. D. Survival of post-autogamous progeny following *LIG4* or *XRCC4* silencing (experiments are independent of the one presented in B). Regen: MAC regenerants. The inserts cloned in plasmids used for feeding experiments are displayed in A. The histogram represents a compilation of several independent experiments, in each of which the progeny of 30 individual autogamous cells was assayed for survival.

To investigate the role of Lig4p during conjugation, *LIG4* expression was knocked down by RNA interference (RNAi), which can be obtained by feeding *Paramecium* cells on bacteria induced for the production of double-stranded RNA (dsRNA) [32]. We used plasmid pLIG4b-L to trigger RNAi against both *LIG4a* and *LIG4b*, which are 93.7% identical within the insert carried by this plasmid (Figure 4A). Reactive cells were obtained by starvation in *LIG4*-silencing medium to ensure mRNA level would be knocked down during meiosis, when it was shown to be the highest. Mating pairs were transferred to standard growth medium and the survival of F1 progeny was scored. Although little lethality was observed in the progeny of *LIG4*-silenced cells relative to a control mating (Figure 4B), genetic analysis indicated that survivors were not produced by the successful development of a zygotic MAC. Indeed, mt7 and mt8 parental cell lines were marked with homozygous recessive alleles of *pwB* and *pwA*, respectively, which both give rise to the same mutant swimming phenotype. Authentic F1 progeny were expected, therefore, to be heterozygous at both loci and to have a dominant wild-type phenotype, as observed in the control (Figure 4B). In contrast, all survivors from *LIG4* silencing exhibited the same mutant phenotype as their parents, as expected if a defect in zygotic MAC development had triggered the regeneration of mutant old MAC fragments [33], [34]. Alternatively, a mutant phenotype could also result from conjugation failure with no exchange of gametic nuclei, giving rise to cells with homozygous mutant MICs and MACs. F1 survivors were therefore submitted to an additional round of autogamy in standard growth medium: most survivors (11/15) produced from *LIG4*-silenced parents, as well as those (14/18) obtained from the control (Zyg mac or Regen. in Figure 4B), gave rise to a fraction of wild-type F2 cells and were therefore identified as real F1 exconjugants. A minority of F1 survivors (4/18 in the control, 4/15 in the *LIG4* knock-down), issued from abortive nuclear exchange (NC in Figure 4B), only yielded mutant progeny. Taken together, these experiments show that *LIG4* silencing during conjugation induces parental MAC regeneration in the progeny, indicative of a strong defect in the development of a functional zygotic MAC. Moreover, because new MICs and MACs originate from the MIC of the previous sexual generation, the ability to produce viable F2 sexual progeny from F1 cells with a regenerated old MAC strongly suggests that *LIG4* silencing during meiosis of conjugating cells has not produced deleterious chromosomal alterations in the zygotic nucleus.

### *LIG4* and *XRCC4* are essential for new MAC development

During conjugation, MAC regeneration is probably favored because there is no active degradation of old MAC fragments when mating pairs are transferred to rich medium [35]. In contrast, during autogamy, cells are kept under prolonged starvation throughout the experiment: this could prevent MAC regeneration due to rapid loss of parental MAC fragments. In *LIG4*-silenced cells, a defect in MAC development might then be monitored by a simple survival assay in the progeny. To test this hypothesis, we injected a constitutive *GFP* transgene into the vegetative MAC of *P. tetraurelia* cells. Injected cells were fed on bacteria producing dsRNA from either of two non overlapping regions of *LIG4* (Figure 4A), then starved to induce autogamy. After 100% autogamy was reached, cells were transferred to standard rich medium to resume vegetative growth (samples I in Figure 4C). A second sample of cells were transferred to rich medium after two additional days of starvation (samples II). Successful formation of a zygotic MAC results in loss of the *GFP* marker contained in the parental MAC, as shown in control experiments. Following *LIG4* silencing, high survival rates were observed when autogamous cells were transferred early to rich medium, but all survivors were fluorescent, indicating that MAC regeneration had occurred. Lethality strongly increased when survival tests were performed after 2 additional days of starvation, consistent with MAC regeneration being prevented by autolysis of old MAC fragments [35].

In all subsequent assays, autogamous cells were kept under prolonged starvation to minimize MAC regeneration, so that we could follow the effect of RNAi on the simple basis of progeny death. Different RNAi plasmids were used to knock down *LIG4a* or *LIG4b* expression: this repeatedly led to massive death of post-autogamous cells (Figure 4C and 4D). In each experiment, MAC regeneration was assayed following starvation of the few survivors in standard medium: *bona fide* sexual progeny were too young to undergo autogamy again, while cells with a regenerated old MAC were able to start a new sexual cycle. The efficiency of each silencing was assayed by quantifying mRNA levels on northern blots. RNAi triggered either by *LIG4a* or *LIG4b* dsRNA resulted in variable decreases in the total amount of *LIG4* mRNA, while a mixture of dsRNA from both genes reduced mRNA levels more significantly (Figure S4). Despite these differences, strong lethality was observed under all conditions in the progeny of silenced cells (Figure 4D). We were concerned that the observed phenotypes could be due to non-targeted silencing of some other transcript by siRNAs produced from *LIG4* RNAi plasmids and, therefore, knocked down the expression of the single-copy *XRCC4* gene, which encodes an essential partner of Lig4p. RNAi against *XRCC4* was found to efficiently reduce its mRNA down to background (Figure S4D) and, like in the previous experiments, strong lethality was observed in the post-autogamous progeny of *XRCC4*-silenced cells (Figure 4D). At the cellular and molecular levels (see below), identical phenotypes were observed upon targeting either *LIG4* or *XRCC4* transcripts.

**Figure 5:**
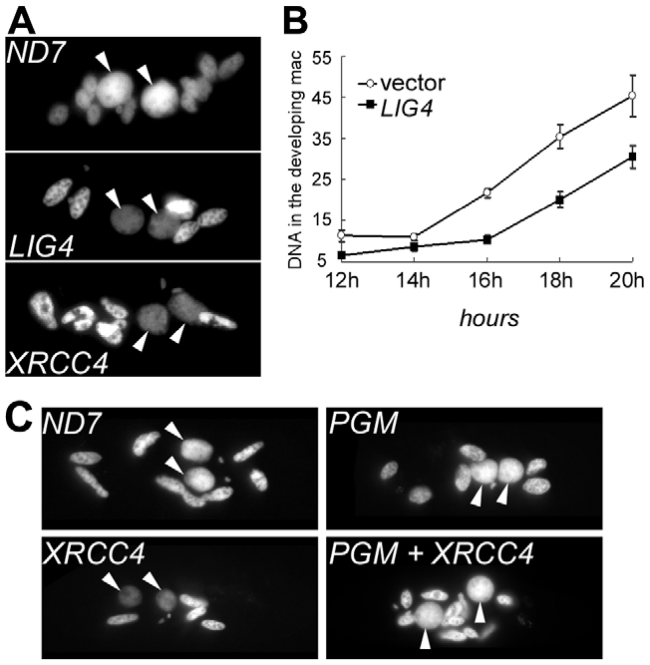
DNA content in developing MACs of *LIG4-* or *XRCC4*-silenced cells. A. DAPI staining of single cells fixed during autogamy in the macronuclear variant 51Δ*A*. Cells were not treated with RNase prior to staining. For *ND7* & *LIG4* silencing, cells were fixed 3 days following transfer to RNAi medium. For *XRCC4,* samples were treated at day 4. White arrows: new MAC. Other stained nuclei are old MAC fragments. B. Quantification of DNA content in propidium iodide-stained exconjugants following RNAi against *LIG4* (pLIG4b-L). Fixed cells were treated with RNase A prior to staining. For each time-point, the average DNA amount (in arbitrary units) contained in the new MACs is displayed in the curve. C. DNA under-amplification in the new developing MACs of *XRCC4*-silenced cells depends on the presence of wild-type levels of PiggyMac transposase. *P. tetraurelia* strain 51 was silenced for the expression of *ND7* (Control), *XRCC4* or *PiggyMac* (*PGM*) individual genes, or cosilenced for *XRCC4* and *PGM*. During autogamy, cells were stained with DAPI and observed using a Zeiss fluorescence microscope (magnification: 630X). Except for the *ND7* control, no viable sexual progeny was recovered from RNAi-treated cells.

RNAi against *LIG4* or *XRCC4* leads either to MAC regeneration or to death of sexual progeny. This suggests that the Lig4p/Xrcc4p complex is essential for the development of a functional new MAC. To monitor MAC development, cells were fixed at different time-points during autogamy, then stained with DAPI. Developing new MACs were observed in *LIG4-* or *XRCC4*-silenced cells but their fluorescence remained very faint, even at late time-points, in sharp contrast to the bright signal observed at similar stages in a control RNAi experiment (Figure 5A). To quantify this difference, we used synchronized conjugation to monitor the total DNA content during MAC development in exconjugants stained with propidium iodide. Exconjugants from *LIG4*-silenced cells often contained smaller new macronuclei relative to the control and quantification revealed consistently lower (∼40%) DNA amounts in their new MACs, indicative either of a replication defect or of DNA degradation (Figure 5B). The characteristic faint staining of the new MACs in Lig4p- or Xrcc4p-depleted cells was dependent on the presence of wild-type levels of the PiggyMac transposase, the putative endonuclease involved in double-strand cleavage of IES boundaries [12], as shown by the normal aspect of developing new MACs in a *PGM*+*XRCC4* double knock-down (Figure 5C). These observations strongly suggest that the Lig4p-Xrcc4p repair complex acts on the PiggyMac-dependent DSBs introduced in the new MAC. DNA underamplification would, therefore, reflect a DSB repair defect in during MAC development.

### Free broken ends accumulate at IES boundaries in *LIG4* or *XRCC4* knock-downs

To analyze the role of Lig4p-Xrcc4p in DNA rearrangements, we examined IES excision intermediates produced during an autogamy time-course of cells submitted to RNAi against *LIG4* or *XRCC4*.

**Figure 6:**
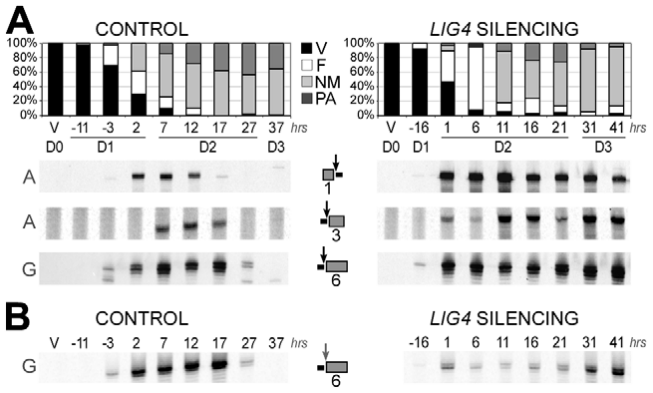
LMPCR detection of free broken ends at IES boundaries in *LIG4*-silenced cells. Three IESs are presented: 1 = 51A1835 (28bp), 3 = 51A4404 (77 bp) from surface antigen *A* gene and 6 = 51G4404 (222 bp) from surface antigen *G* gene. Gene names are indicated on the left of panels A and B. IESs are drawn as grey boxes and MAC flanking sequences as black lines. Vertical arrows indicate the position of DNA cleavage in each experiment. All details about the linkers and primers used in this experiment are provided in Table S1. In both time-courses, cells were transferred to RNAi medium at day 0 (D0). The T0-time point is the time when 50% of cells have a fragmented old MAC. RNAi against *LIG4* was obtained using mixed bacterial cultures producing dsRNA from *LIG4a* (pLIG4a-R) and *LIG4b* (pLIG4b-R). Histograms show the progression of autogamy. V: vegetative cells. F: fragmented parental MAC. NM: cells with two visible new developing MACs. PA: post-autogamous cells with one MAC and surrounding fragments. A. LMPCR detection of free broken MAC ends at IES excision sites during autogamy of strain 51 submitted to control or *LIG4* RNAi. B. LMPCR detection of free broken IES ends at the left boundary of IES #6 during the same time-course.

**Figure 7:**
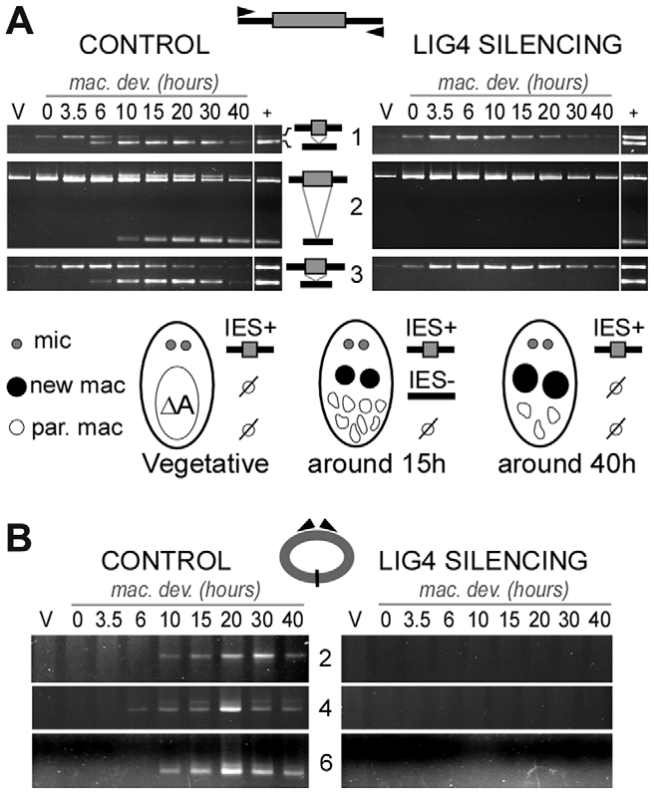
Absence of final junction products following IES excision in *LIG4*-silenced cells. IESs #1 ( = 51A1835), 3 ( = 51A4404) and 6 ( = 51G4404) are described in the legend to Figure 6. Two additional IESs from surface antigen *A* gene are presented: 2 = 51A2591 (370 bp) and 4 = 51A4578 (883 bp). In each diagram, IESs are represented by a grey box and their flanking MAC sequences by a black line. PCR primers are drawn as arrowheads. A. Detection of IES excision junctions in the *A* gene in the macronuclear variant 51Δ*A*. Top: gel electrophoresis of PCR products around IESs. Bottom: representation of MAC development stages. In vegetative cells, only the MIC contributes to the PCR signal (IES+). When new MACs develop, *de novo* chromosomal junctions give an IES- PCR signal. At later stages, this signal disappears due to maternal epigenetic inheritance of the *A* deletion [13]. B. Detection of IES circle junctions by PCR in 51Δ*A*, using two internal divergent primers for each IES (same time-course as in A).

Total genomic DNA was extracted from vegetative cells and at different time-points during MAC development. We first used LMPCR to detect DSBs at IES boundaries during autogamy of strain 51. Free broken chromosome ends were visualized at early time-points during MAC development of cells submitted to a control RNAi, but they were only transient, indicative of efficient closure of IES excision sites (Figure 6A). In cells silenced for *LIG4*, DSBs started to accumulate at the same autogamy stage but broken ends were found to persist until the last time-points, both on the flanking sequences that should be joined to form MAC DNA, which will be designated as MAC ends (Figure 6A), and at IES ends (Figure 6B). These observations support the hypothesis that *LIG4* silencing results in a defect in DSB repair, downstream of DNA cleavage. Therefore, we followed the closure of IES excision sites during autogamy, using strain 51Δ*A,* a macronuclear variant of strain 51, which harbors a wild-type MIC genome but carries a deletion of the surface antigen *A* gene in its MAC [13]. We took advantage of the fact that, during autogamy of 51Δ*A*, the *A* gene (absent from the parental MAC) is transiently amplified in the new developing MAC, before being deleted at later stages, making it possible to monitor the formation of *de novo* excision junctions by a simple PCR assay. Such junctions were readily amplified from control cells but none could be detected in *LIG4*- (Figure 7A) or *XRCC4*-silenced cells (Figure S7A), indicating that the precise closure of IES excision sites on MAC chromosomes requires Lig4p-Xrcc4p. In these experiments, we confirmed by LMPCR that free broken ends accumulate at IES boundaries in 51Δ*A* cells silenced for *LIG4* (Figure S6) or *XRCC4* (Figure S7B). Interestingly, specifically for IESs from the *A* gene, DSBs with the expected 5′ overhangs were introduced normally at early time-points but could not be detected at later time-points (Figure S6), perhaps as a consequence of maternal inheritance of the *A* gene deletion. With regard to excised molecules, we reported previously that IESs of sufficient length are circularized after excision [16]. We used divergent primers internal to some IESs to monitor the formation of covalently closed excised circles by PCR (Figure 7B). Here again, no junction products could be detected in cells silenced for *LIG4*, indicating that end joining is also required for IES circularization.

### Lig4p-Xrcc4p is dispensable for DSB 5′ processing but is required for nucleotide addition to 3′ ends

DSB processing during autogamy is thought to occur before the final joining of chromosome ends at IES excision sites [11]. Processing includes the removal of the 5′ terminal nucleotide and the addition of one nucleotide to the 3′ end of the break.

**Figure 8:**
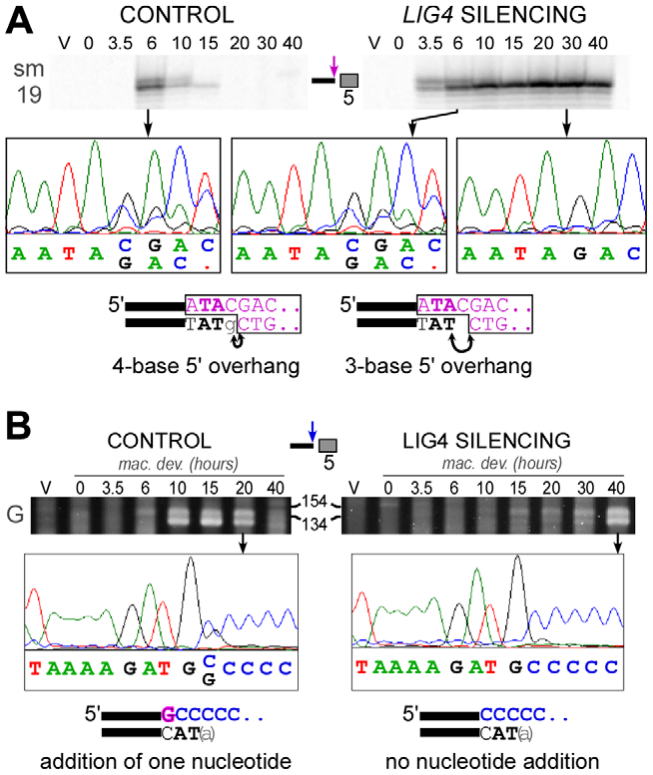
Analysis of 5′- and 3′-end processing at double-strand breaks in *LIG4*-silenced cells. A. LMPCR detection of 4-base and 3-base 5′ overhangs on the broken MAC ends generated at the left boundary of IES #5 = sm19-576 (66 bp) during autogamy of strain 51Δ*A* submitted to control or *LIG4* RNAi. LMPCR products were separated on high resolution polyacrylamide denaturing gels to visualize the doublet of bands. The position of linker ligation to the free 5′ end was confirmed by gel purification of LMPCR products and sequencing using primer sm19-4 specific for the left flanking MAC sequences (the chromatograms show the sequence of the top strand). The structure of the ligation products is diagrammed at the bottom, with the linker represented in purple. Arrows indicate the nucleotides that are ligated to the linker on 4-base or 3-base 5′ extensions. B. Nucleotide addition to broken MAC 3′ ends is impaired in Ligase IV-depleted cells. Terminal transferase-mediated poly(C) tailing of the MAC left 3′ end of IES #6 (51G4404) was performed as indicated in [11]. Poly(C)-tailed products were amplified using primers 51G18 and I, then a nested PCR was performed with 51G13 and I before electrophoresis on a 3% Nusieve agarose gel. The position of size standards (in bp) is indicated: PCR products are expected around 122 bp, with some variability due to the length of the poly(C) tail added by the terminal transferase. To determine the position of poly(C) addition, gel-purified PCR products were sequenced using 51G13 (from the MAC left flanking sequences) as a sequencing primer. The chromatograms show the sequence of the top strand and the structure of the broken MAC ends identified in each sample is displayed at the bottom.

To analyze 5′ end processing, LMPCR products obtained following *in vitro* ligation of a linker to the free 5′ overhangs carried by broken MAC ends, can be resolved at the nucleotide scale on denaturing polyacrylamide gels (Figure 8A). This makes it possible to distinguish a doublet of bands, clearly visible at early-time points (6 hrs) in a control autogamy time-course and during autogamy of *LIG4*-silenced cells. Only the bottom band was found to accumulate until the last time-point (40 hrs) in cells depleted for Lig4p. The linker used in this experiment carries a nonphosphorylated 5′-ATAC overhang that guides linker ligation to the 4-base 5′ extension generated at the broken MAC end by initial PiggyMac-dependent cleavage. As previously reported [11], *in vitro* ligation may also occur through a 1-nt gap, which would covalently join the linker to a processed 5′ end, from which the 5′ terminal nucleotide has been removed (see diagrams at the bottom of Figure 8A). The presence of 4-base and 3-base 5′ overhangs was confirmed by DNA sequencing of the doublets observed at early time-points, while only 3-base overhangs accumulate at later time-points in *LIG4*-silenced cells. We can conclude, therefore, that Lig4p is not required for normal removal of the 5′ terminal nucleotide from broken MAC chromosome ends.

To investigate whether the polymerizing step still takes place in *LIG4* knock-downs, we used terminal transferase-mediated poly(C)-tailing to map the position of free 3′ ends at broken MAC ends. For the two IESs presented in this study, we detected only the 3′ end generated by initial PiggyMac-dependent cleavage when cells were depleted for Lig4p, in contrast to the control, in which the poly(C) tail was branched at the expected two positions (Figure 8B and Figure S8). This result indicates that nucleotide addition to broken 3′ ends is strongly impaired in *LIG4*-silenced cells.

## Discussion

### IES excision: a “cut-and-close” mechanism

Ever since developmentally programmed genome rearrangements were reported in ciliates, identifying the key enzymes that catalyze DNA elimination has constituted a challenge. DNA rearrangements were shown to be maternally controlled *via* non-coding RNAs and a specialized RNA interference pathway, which mediate genome-wide comparison of maternal MIC and MAC genomes and guide elimination of MIC-restricted sequences from the new MACs (reviewed in [36], [37]). Previous screens for indispensable IES excision genes in *P. tetraurelia* uncovered the essential role of a developmentally regulated SUMO pathway likely to operate in the developing new MAC [34] and of Die5p, a nuclear protein of unknown function acting at a late step during DNA rearrangements [38]. The recent discovery that domesticated transposases are essential for initial DNA cleavage in *Paramecium* and *Tetrahymena* has provided strong evidence that IES excision is related to cut-and-paste transposition [12], [39]. In *Oxytricha,* a more distant ciliate, transposases from another family have also been implicated in DNA rearrangements [40].

Our data point to an absolute requirement for Lig4p and Xrcc4p in IES excision, downstream of PiggyMac-dependent cleavage of IES ends. Consistent with a direct participation in DNA rearrangements, a Lig4p-GFP fusion protein accumulates in developing new MACs by the time IES excision takes place. Most IESs studied in this work are cleaved at the same developmental stage in *LIG4*-silenced cells as in the control, suggesting that depleting Lig4p does not interfere directly with DNA cleavage. One notable exception is IES 51G4404, for which a delay in DSB introduction was conspicuous, both by poly(C) tailing (Figure 8B) and by LMPCR analysis of DSBs (Figure S6), in autogamy time-courses with a particularly good synchrony. However, this difference was not observed in all cultures and additional work is needed to evaluate the generality of this observation.

**Figure 9:**
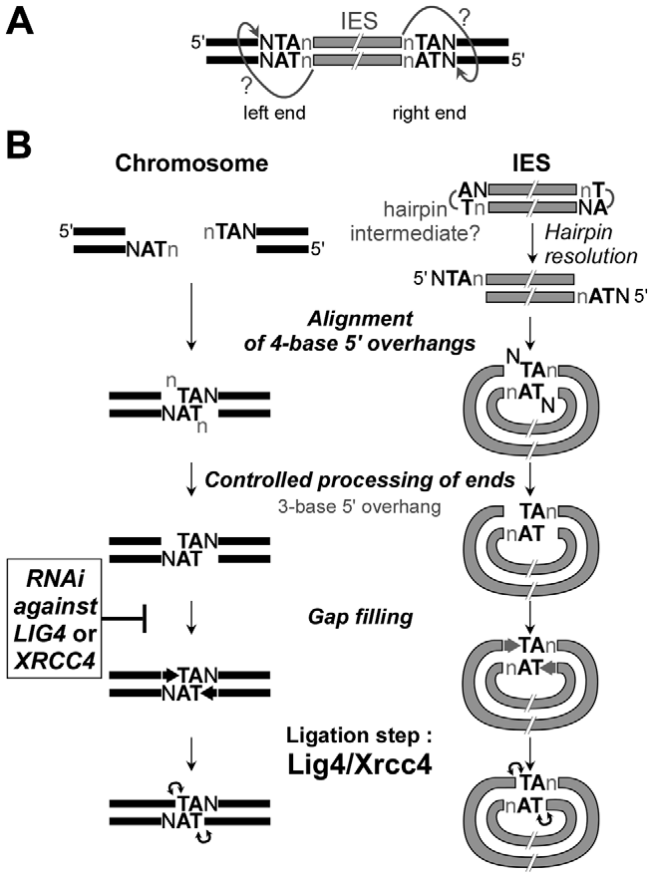
Model for IES excision in *P. tetraurelia.* A. Model for initial cleavage at IES boundaries by the PiggyMac-associated complex. Nicking of the first strand would liberate a reactive 3′OH residue at IES ends. The putative nucleophilic attack of the top strand is represented by arrows with question marks. B. DNA intermediates and end joining factors involved in IES excision are shown. In wild-type conditions, repair of the chromosomal excision sites (left) is probably mediated by alignment of 4-base 5′ overhangs *via* the pairing of their central conserved TA. The formation of IES circles (right) may require the resolution of putative hairpins at IES ends, prior to the formation of intramolecular paired-end intermediates. The actors involved in the controlled processing of ends are still unknown. At least for the MAC chromosome junctions, the removal of the 5′ terminal nucleotide does not require Lig4p/Xrcc4p, in contrast to the gap filling step, which is strongly inhibited in *LIG4* knock-downs. The final ligation is totally Lig4p/Xrcc4p-dependent. In *LIG4* or *XRCC4* silencing, no MAC junctions are formed and DSBs accumulate. No circle junctions could be detected.

Both the precise closure of excision sites on MAC chromosomes and the circularization of excised IESs require the Lig4p-Xrcc4p end-joining complex. Formally, our data may still fit with a model in which cleavage at one IES boundary would initiate excision, while second-end cleavage would be impaired in cells depleted for Lig4p or Xrcc4p. Single-end cleaved IESs still attached to their flanking MAC sequences at their other end can indeed be detected by LMPCR during the course of autogamy in standard medium [13], or by the tailing of 3′ ends in control or *LIG4* knock-downs (Figure S8B). However, they appear only transiently and do not accumulate in Lig4p-depleted cells, in contrast to DSBs at flanking MAC-destined ends (Figure S8A and S8B). Furthermore, LMPCR experiments using a linker compatible with both ends of one particular IES (51G4404) allowed the detection of larger amounts of excised linear molecules in *LIG4*-silenced cells than in a control RNAi (Figure S8D). These observations rule out the participation of direct DNA transesterification in assembly of chromosome and circle junctions and rather support a model, in which two double-strand cleavages, one at each end, initiate IES excision ([11], see Figure 9A). We propose that Lig4p, in association with Xrcc4p, precisely joins the two resulting broken ends on MAC chromosomes (Figure 9B). Similarly, covalently closed circles are secondary products of the reaction and their formation also depends on Lig4p-Xrcc4p: for those IESs flexible enough to bring their ends together, circularization would prevent reactive 3′OH groups generated by DNA cleavage at IES ends from transposing to other target sites in the genome. Thus, in contrast to cut-and-paste transposition, IES excision uses a “cut-and-close” mechanism, in which developmentally programmed end joining efficiently drives somatic chromosome assembly and avoids reintegration of excised sequences.

In the macronuclear variant 51Δ*A*, which carries a macronuclear deletion of the *A* gene, we observed that, specifically for IESs carried by the *A* gene, DSBs were introduced normally, but they diminished over time (Figure S6), concomitantly with the deletion of the *A* gene. Notably, macronuclear deletion of the *A* gene is associated with chromosome fragmentation, with telomere addition at heterogeneous positions upstream of the gene transcription start [41]. This points to a possible mechanistic difference between IES excision and chromosome fragmentation, and suggests that the breaks that cause imprecise elimination of the *A* gene are not protected, leading to degradation of the whole region.

### DSB repair and DNA replication during MAC development

The transcription of *LIG4* and *XRCC4* is induced during meiosis, largely before programmed genome rearrangements take place in developing new MACs. This pattern led us to consider the possibility that NHEJ proteins may be involved in the repair of meiotic DSBs and that a depletion in Lig4p-Xrcc4p could induce deleterious genome rearrangements during MIC meiosis, which would be transmitted to the new MICs of the progeny and strongly impinge on normal development of the new MACs. However, we checked the progression of meiosis by DAPI staining during autogamy of cells silenced for *LIG4* or *XRCC4* expression and observed that meiotic divisions I and II occur normally (Figure S5), with no arrest in meiosis until new MACs differentiate from mitotic copies of the zygotic nucleus. Furthermore, genetic analysis of the progeny of conjugating cells silenced for *LIG4* indicated that the new MICs of the following sexual generation were fully functional germline nuclei (see Figure 4B and related text). We did observe a novel phenotype in the new MACs that develop in cells silenced for *LIG4* or *XRCC4*: quantitative analysis revealed an anomalously low DNA content within these nuclei (Figure 5). Strikingly, this phenotype was suppressed by a double RNAi targeting both *PiggyMac* and *XRCC4*, suggesting that it does not result from a meiotic defect but, rather, from a global replication slow-down caused by the accumulation of PiggyMac-dependent DSBs in the developing new MACs, or from active DNA degradation at unrepaired broken ends. Persistent DSBs with the expected geometry could be detected at IES ends using a sensitive LMPCR assay, even at late time-points. This suggests that broken ends are, at least in part, protected against extensive degradation in cells depleted for Lig4p or Xrcc4p and favors the idea that unrepaired DSBs at IES excision sites would block the progression of DNA replication. In spite of all our efforts, we have been unable to detect any degradation products by Southern blot hybridization of total genomic DNA (not shown), consistent with the idea that broken chromosomes are under-amplified.

### Free, but incompletely processed broken ends accumulate at IES boundaries in *LIG4* or *XRCC4* knock-downs

Like the PiggyMac domesticated transposase, the Lig4p-Xrcc4p complex is an essential component of the IES excision core machinery. In our “cut-and-close” model for IES excision, the final ligation step performed by the Lig4p-Xrcc4p complex is thought to take place within a paired-end intermediate (Figure 9B), in which the two 4-base 5′ overhangs generated by initial cleavage at each IES boundary are aligned *via* the pairing of their central conserved TA dinucleotides. In this intermediate, the 5′ terminal base of each broken end is generally not complementary to its facing nucleotide and, as reported earlier, highly controlled removal of these mismatched bases by yet unknown nuclease activities, and gap filling by addition of one nucleotide to the free 3′ end precede the final ligation step [11]. Interestingly, close examination of LMPCR products separated on high-resolution denaturing gels revealed that most persisting broken ends in *LIG4*-silenced cells carry a 3-base 5′ overhang (Figure 8A and data not shown). The conversion of 4-base to 3-base overhangs is thought to reflect the removal of the 5′-terminal nucleotide during the repair of IES excision sites ([11], see Figure 9B). Our observation, therefore, suggests that controlled 5′-processing of broken ends still takes place in cells depleted for Lig4p-Xrcc4p. In contrast, our data point to an inhibition of the polymerizing reaction that carries out the addition of one nucleotide to the free 3′ end prior to the ligation step (Figure 8B and Figure S8C). Our *in vivo* data provide further support to the idea that polymerase activity (or recruitment) during NHEJ repair is strongly dependent on Lig4p-Xrcc4p, a hypothesis previously proposed by others, based on observations made in a cell-free NHEJ system [42].

Another implication of our results is that the processed chromosome ends appear to be protected against extensive resection, as judged by the accumulation of LMPCR products observed in cells depleted for Lig4p. However, for a given IES boundary, we noticed an asymmetry in the amounts of broken ends detected on the IES side (ends of excised molecule) and on the MAC side (flanking the excision site). Even though LMPCR assays are not quantitative, free IES ends were consistently detected in lower amounts in *LIG4*-silenced cells relative to control, while their detection level still increased at late time-points (Figure 6B). Two non-mutually exclusive hypotheses may account for this asymmetry. On the first hand, as shown for a canonical *piggyBac* transposase [43], PiggyMac may introduce hairpins at the ends of excised IESs (Figure 9), which would not be detected in our LMPCR assay. These hairpins might be less efficiently converted to 5′ overhangs if recruitment of Lig4p-Xrcc4p were required to stabilize a resolution complex. On the other hand, broken MAC ends, but not IES ends, may be strongly protected against resection, even in the absence of Lig4p-Xrcc4p. Such protection could be mediated by the PiggyMac-associated cleavage complex itself. Alternatively, the Ku70/Ku80 heterodimer, which was shown by others to control the precision of the NHEJ pathway by inhibiting DSB resection [44], could also contribute to protecting chromosome ends. *P. tetraurelia* harbors several *KU* genes in its genome, some of which are specifically induced during MAC development (Figure S3), and future work will elucidate their role in IES excision.

### Highly precise NHEJ and a domesticated transposase participate in developmentally programmed genome rearrangements

Our work provides strong evidence that the Lig4p-Xrcc4p complex, a key actor of the NHEJ pathway, carries out DSB repair during developmentally programmed IES excision in *Paramecium*. End joining is highly precise at the nucleotide level and this is critical for the assembly of functional open reading frames in the somatic genome. Several observations support the idea that tight coupling between DNA cutting and repair contributes to this precision. The early induction of *LIG4* and *XRCC4* genes, well before new MACs start to develop, indicates that their expression is developmentally programmed rather than triggered by DNA breaks: thus, the preexisting end-joining ligation complex could readily be recruited to DSBs as soon as they appear in the new developing MACs. Moreover, we have proposed that IES ends form a synapse before they are cleaved [13], perhaps as a result of binding by the PiggyMac domesticated transposase. If not dissociated following DSB introduction, this synaptic complex could keep broken ends together for subsequent repair. In addition, the conservation of TA dinucleotides at IES ends, shown to be essential for DNA cleavage [13], may also contribute to precise repair. Indeed, all broken ends generated at IES boundaries exhibit the same characteristic geometry, with 4-base 5′ overhangs carrying a central TA that could guide their partial pairing.

IES excision starts after a few rounds of genome amplification have taken place in the developing MAC and 16 to 32 copies of each germline chromosome may be present when the first DSBs are introduced [16]. At least for the first excision events, we believe that NHEJ, which has long been referred to as error-prone, is the major pathway involved in the closure of excision sites. Homologous recombination is unlikely to account for these first events, since the new MAC differentiates from a copy of the germline nucleus, which only harbors the nonrearranged version of the genome: therefore, no rearranged template DNA is expected to preexist in the new MAC when it starts to develop. Thus, like V(D)J recombination in vertebrates, IES excision is a developmentally programmed DNA elimination relying on a domesticated transposase for the introduction of initiating DSBs and on the NHEJ pathway for the joining of flanking DNA ends. However, the two systems differ strikingly with regard to precision, V(D)J recombination joints being largely variable [4]. Our results support the notion that the diversity of the chromosomal junctions generated by V(D)J rearrangements does not result from inherent imprecision of the NHEJ pathway. An alternative explanation may reside in differences in the structure of the broken ends generated in the two systems or in their different processing. Rag1/Rag2-mediated cleavage generates blunt ends on the excised intervening fragment, which are ligated precisely to form a signal joint, while DNA hairpins are formed at flanking chromosome ends, which are opened in an imprecise manner, processed *via* nucleotide loss and/or addition before being joined by Lig4p-Xrcc4p. In contrast, cleaved ends generated in a PiggyMac-dependent manner during IES excision can readily align and pair, and require only limited processing before the final ligation step. *Paramecium*, therefore, provides a novel example of an essential role of NHEJ in the precise repair of developmentally programmed DSBs at a genome-wide scale.

## Materials and Methods

### Bioinformatics and phylogeny

*LIG* and *XRCC4* genes were identified in the macronuclear genome of *P. tetraurelia* by BLAST searches at ParameciumDB (http://paramecium.cgm.cnrs-gif.fr/) [45]. For DNA ligases, we first selected the genes that blasted against ligase genes from other organisms in the automated annotation of the MAC genome sequence [17]. In parallel, we used human ligases for tblastn search on the *Paramecium* genome. Finally, all putative *Paramecium* sequences were blasted against the whole genome to ensure that no paralog would be omitted and manual curation of gene annotations was carried out. As a query sequence for Xrcc4p, we used the 23-aa peptide from the human homolog, which was shown previously to interact with Ligase IV [46].

Phylogenetic trees were constructed using MEGA 4 Neighbor-Joining algorithm [47]. For Figure 1, we used a ClustalW alignment obtained from the NPS server [48].

### *P. tetraurelia* strains and growth conditions

For autogamy time-course experiments, we used *P. tetraurelia* strain 51 (51 new in [11]) and its 51Δ*A* variant carrying a heritable deletion of the *A* gene in its MAC but harboring a wild-type MIC [13]. Cells were grown at 27°C in a wheat grass infusion (WGP; Pines International Inc.) inoculated with *Klebsiella pneumoniae* as described [16]. Autogamy was monitored by 4′-6-diamidino-2-phenylindole (DAPI)-staining. Total RNA and genomic DNA were extracted from ∼400,000 cells for each time-point, as described [12]. Genomic DNA was quantified using the QuBit assay kit (Invitrogen) and RNA concentrations were estimated by absorption at 260 nm.

Derivatives of strain 51 were used for conjugation experiments. For genetic analysis, we used strain a3093 (mt7) homozygous for *pwB-96* and *nd9-c* and strain d4-502 (mt8) homozygous for *pwA-502* and *nd6-1* (kindly supplied by Mihoko Takahashi, University of Tsukuba). Cells were grown at 27°C in a pea medium inoculated with *K. pneumoniae* as described [34].

*P. tetraurelia* stock d4-110 (*hr-b*/*hr-b*) was used for synchronized conjugation: following mixing of reactive cells, conjugating pairs were synchronized and concentrated using iron dextran particles and strong neodymium magnets [34]. Total RNA was isolated from 50- to 100-mL aliquots of *Paramecium* cell culture (100 to 1,000 cells/mL), using the RNeasy Mini kit (QIAGEN) supplemented by a QIA shredder (QIAGEN) for homogenization and the RNase-free DNase set (QIAGEN) for genomic DNA elimination [34].

For localization of the Lig4p-GFP fusion, plasmid pLIG401GC was injected into the MAC of vegetative a3093. It contains the 496-bp genomic region upstream of the *LIG4a* open reading frame, the whole *LIG4a* open reading frame with the green fluorescent protein (GFP) gene fused to its 3′ end and the 3′UTR of gene *G^156^* from *P. primaurelia* (sequence available upon request). Plasmid pRB35, containing the wild-type *pwB* gene with its 114-bp upstream and 233-bp downstream regions, was used to screen for successfully injected cells based on the complementation of the *pwB* phenotype and injected cells were mated with reactive d4-502 (*pwA*). A few hours after pair separation, two sub-populations could be sorted out according to their swimming phenotype when transferred to 20 mM KCl-containing medium. Exconjugants from injected cells exhibited a wild-type phenotype and swam backward for >30 secs, while those from *pwA* mutants swam backward slower and for shorter times. Details of GFP fluorescence analysis are provided in Text S1.

### Molecular procedures

Oligonucleotides were purchased from Sigma-Aldrich or Eurofins MWG Operon (see Table S1).

PCR amplifications were performed in a final volume of 25 µL, with 10 pmol of each primer, 5 nmol of each dNTP and 1 U of DyNAzyme II DNA polymerase (Finnzymes), using an Eppendorf Mastercycler personal thermocycler. PCR products were analyzed on 3% NuSieve GTG agarose gels (BioWhittaker Molecular Applications). LMPCR detection of double-strand breaks was performed as described [13]. Terminal transferase was used for poly(C) tailing of free 3′ ends and, following synthesis of the complementary strand from the Anchor(G) primer, tailed products were amplified by PCR as described [11]. All DNA sequencing was performed by GATC Biotech.

Northern blot and dot-blot hybridization were carried out as described in [12] for autogamy time-course experiments and in [34] for conjugation experiments.

### RNA interference by feeding

#### RNAi plasmids

All RNAi plasmids are derivatives of vector L4440 [49] and carry a target gene fragment between two convergent T7 promoters (see Text S1 for a detailed description).

#### RNAi during conjugation

*P. tetraurelia* reactive cells (mt7 *pwB nd9* and mt8 *pwA nd6*) were obtained by feeding on *Escherichia coli* bacteria producing double-stranded RNA from plasmid pLIG4b-L [34]. Conjugation of RNAi-treated cells was induced within 48 hrs after inoculating the culture with *E. coli*. Conjugating pairs were transferred to standard *K. pneumoniae* medium and allowed to grow. For genetic analysis, F1 exconjugants from each pair were separated and grown for about 10 cell divisions before phenotypes were scored. Wild-type swimming behavior was indicative of successful conjugation, while a mutant phenotype in F1 revealed either conjugation failure or parental MAC regeneration. To distinguish between the last two possibilities, about 10 cells from each starved F1 were transferred to standard medium and allowed to grow before undergoing autogamy. F2 progeny of cells issued from a failed conjugation event should all exhibit a mutant phenotype, while 25% wild-type cells are expected in the post-autogamous progeny of MAC regenerants.

During conjugation, cells were fixed and treated with RNase and nuclear DNA was stained by VECTASHIELD with propidium iodide (see Text S1).

#### RNAi during autogamy

RNAi during autogamy was performed on strains 51 or 51Δ*A* as described [12]. At day 1 of starvation, cells were generally 100% autogamous and survival of their progeny was tested at day 2 or day 4 by transferring 30 individual autogamous cells to standard *K. pneumoniae* medium.

To monitor MAC regeneration, strain 51 was injected with *Bgl*I-restricted pZC'ΔRI, a modified pUC vector carrying the GFP-coding sequence adapted to *Paramecium* codon usage under the control of *P. primaurelia G^156^* transcription signals (sequence available upon request). Following autogamy, GFP fluorescence was observed on living cells under a Leica fluorescence binocular. Presence of the GFP transgene in regenerated MACs was confirmed by dot-blot hybridization of total genomic DNA with a ^32^P-labeled GFP probe.

## Supporting Information

Figure S1 Phylogenetic tree of ATP-dependent DNA ligases. Amino-acid sequences of 61 ATP-dependent DNA Ligases were aligned using the MUSCLE algorithm [50]. Non-informative regions were removed manually from the alignment. The phylogenetic tree was generated by neighbor-joining using MEGA 4, with the following parameters: bootstrap 1000, pairwise deletion of gaps, equal input model, heterogeneous pattern among lineages, gamma distributed rates among site (with a gamma shape parameter  =  1.7, as estimated by the means of gamma parameters calculated with the PhyML algorithm at http://www.hiv.lanl.gov). Bootstrap values are not shown when equal to 100. Prokaryotic NAD+-dependent replicative ligases (LigA) are not included. Some bacteria possess ATP-dependent DNA ligases (Ligases C or D) involved in DNA repair [51], [52]. Some also encode ATP-dependent type K Ligase homologues: 3 randomly selected bacterial LigK are represented. ParameciumDB accession numbers of *Paramecium* genes are provided in the legend to Figure 1. TtL1a = XP_001022972.1 on GenBank. For all other proteins, Uniprot accession numbers are: TtL1b = Q24FD9, TtL4 = Q23RI5, TtlK = Q233G4, LmaL1 = Q4Q6U5, LmaKa = Q4Q960, LmaKb = Q4Q959, TbrL1 = Q587E4, TbrKa = Q6V9I8, TbrKb = Q56AN9, TcrL1a = Q4DX91, TcrKa = Q4DMH8, TcrKb = Q4DMH7, AtL1 = Q9C9M5, AtLIG1 = Q42572, AtL4 = Q9LL84, HsL4a = P49917, MmL1 = P37913, MmL4 = Q8BTF7, SpL = Q9C1W9, SpL4 = O74833, MkLb = Q8TWN3, MmeL1 = A6VFQ9, VvL = P33798, AfLD = O28549, AfLB = O29632, AtuLDa = A9CLR5, Cj = Q5HSC4, BpLD = Q63I59, HiL = P44121, MaLC = Q744K0, MaLD = Q742F5, MlLC = Q98NY5, MlLDa = Q98DP8, Nm = C6S4M8, PaLD = Q9I1X7, SwLD = Q0AXX1.
(TIF)

Figure S2 Conservation of the amino acids responsible for the Xrcc4–DNA ligase IV interaction. A multiple sequence alignment of the Lig4p-interacting region of Xrcc4p homologs from different organisms is displayed in the top panel. The linker regions connecting the BRCT domains of Lig4p homologs and involved in the interaction with Xrcc4p, are aligned in the bottom panel. Amino acids (aa) involved in the interaction between human ligase IV and Xrcc4p are shown (|). Strongly conserved residues are in cyan, identical in bold and red, and essential residues are highlighted in yellow [46]. Hs: *Homo sapiens*, At: *Arabidopsis thaliana*, Dm: *Drosophila melanogaster*, Sc: *Saccharomyces cerevisiae*, Pt: *Paramecium tetraurelia*.
(TIF)

Figure S3 Expression of *P. tetraurelia* DNA ligase and NHEJ genes during autogamy. NimbleGen whole-genome microarrays carry 6 oligonucleotide probes per gene: each slide was hybridized with a cDNA sample and the median of the six signals was calculated ([29], data available in ParameciumDB). Because all oligonucleotide probes present on the slides do not have the same Tm, microarrays do not allow comparing absolute transcript levels for different genes and only provide information on the relative variations of expression for each individual gene during autogamy. Following clusterization of slides, autogamy stages were defined as follows: VEG, vegetative cells; MEI, MIC meiosis; FRG, fragmented old MAC and no detectable new MAC following DAPI staining; DEV, visible developing new MACs (1, 2, 3 refer to three successive stages). Expression profiles are drawn from the mean values obtained for each stage from 4 independent time-course experiments. A. *Paramecium* ATP-dependent DNA ligases. For the particular case of *LIG4b*, automatic annotation of the draft MAC *P. tetraurelia* genome has split the gene into two open reading frames (GSPATG00022021001 and GSPATG00022020001). Thus, two sets of 6 probes contribute to the *LIG4b* signal and the curve represents the median of all 12 probes for each stage. This analysis reveals that two out of three *LIG1* genes are induced early during sexual processes, as expected for a postulated role in DNA replication, during meiosis or MAC development. The four *LIGK* are strongly upregulated at later stages during MAC development: future work should provide more insight into their function. B. Core NHEJ genes present in the *P. tetraurelia* genome. *XRCC4* accession number in ParameciumDB can be found in the legend to Figure 4A. Other ParameciumDB accession numbers are: GSPATG00006445001 for *KU70a*, GSPATG00009747001 for *KU70b*, GSPATG00034664001 for *KU80a*, GSPATG00035446001 for *KU80b* and GSPATG00030095001 for *KU80c*. Putative *CERNUNNOS* (*CER*) genes are also represented. Identification of *CER* genes (I. Callebaut, pers. comm.) was carried out by PSI-BLAST search and Hydrophobic Cluster Analysis (HCA) [53]–[55], as described in [56]. Both *CERa* and *CERb* were reannotated following manual curation: accession numbers in ParameciumDB are PTETG200014001 and PTETG2200011001, respectively.
(TIF)

Figure S4 Quantification of *LIG4* and *XRCC4* mRNA levels in silenced cells. Northern blots of total RNA extracted during different autogamy time-courses were probed with *LIG4* (A, B & C) or *XRCC4* (D) probes. All values were normalized with 17S rDNA signal for quantification. VK corresponds to vegetative cells grown in standard bacteria before transfer to silencing medium. The t = 0 hrs time-point was set as the time when 50% of cells had a fragmented old MAC. Times are in hours. A. RNAi was carried out on strain 51 (same experiment as in Figure 6), using pLIG4b-L (Figure 1) to induce the production of *LIG4* dsRNA. Total RNA samples extracted from both *LIG4*- and *ND7*-silenced cells were blotted onto a single membrane and hybridized to a *LIG4* probe. Time-points of *ND7* and *LIG4* RNAi experiments are displayed separately. B. RNAi against both *LIG4* genes was performed on strain 51Δ*A* (same experiment as in Figure 7 and Figure S6), using pLIG4a-R and pLIG4b-R (Figure 4A) for dsRNA production. Samples from *LIG4* and *ND7* silencing experiments were transferred separately to two different northern blots and the VK sample was loaded in duplicate on each membrane (1: *LIG4* blot; 2: *ND7* blot). The two blots were hybridized together with a *LIG4* probe. C. Three different dsRNA-producing constructs were tested separately to induce RNAi against *LIG4* genes in strain 51Δ*A*. All samples were loaded on the same blot and hybridized with a *LIG4* probe. Silencing using pLIG4a-R or pLIG4b-R was found to be as efficient as with pLIG4b-L, based on northern blot hybridization and on high lethality rates in the progeny of silenced cells. Autogamy stages are displayed below each diagram. V: vegetative cells; M: MIC meiosis; F: fragmented old MAC; NM: two visible new developing MACs; PA: post-autogamous cells with one MAC and surrounding fragments. D. RNAi against *XRCC4* was applied to strain 51Δ*A* using plasmid pXRCC4-L (Figure 4A). RNA samples from *XRCC4*- and *ND7*-silenced cells (same experiment as in Figure S7) were transferred to two separate membranes but hybridized together with an *XRCC4* probe. VK sample was loaded on both membranes (1: *LIG4* blot; 2: *ND7* blot). St  =  starved (St1N  =  -11 hrs; St2N  =  -4 hrs; St1X  =  -13 hrs; St2X  =  -1,5 hrs).
(TIF)

Figure S5 Normal progression of meiosis during the silencing of *LIG4* or *XRCC4*. DAPI staining of single cells fixed during autogamy in the macronuclear variant 51Δ*A*. Cells were not treated with RNase prior to staining. During *LIG4* silencing (A, C & D), starved cells were fixed 1 day following transfer to RNAi medium. For *XRCC4* silencing (B), the sample was treated at day 2. White arrows: micronuclear meiotic products. The heavily stained nucleus in each panel corresponds to the MAC. A. First meiotic division. B, C and D. After meiosis II. The eight haploid mics are sometimes not all visible, when they are not in the same focus.
(TIF)

Figure S6 LMPCR detection of DSBs during autogamy of the macronuclear variant 51Δ*A* silenced for *LIG4*. Four IESs are presented: 1 = 51A1835 (28 bp), 3 = 51A4404 (77 bp) from surface antigen *A* gene; 5 = sm19-576 (66 bp) from *SM19* tubulin gene and 6 = 51G4404 (222 bp) from surface antigen *G* gene. Gene names are indicated on the left of panels A and B. MAC flanking sequences are drawn as black lines and IESs as grey boxes. Vertical arrows indicate the position of DNA cleavage in each experiment. In all time-courses, cells were transferred to RNAi medium at day 0 (D0). The T0-time point is the time when 50% of cells have a fragmented old MAC. RNAi against *LIG4* was obtained using mixed bacterial cultures producing dsRNA from *LIG4a* (pLIG4a-R) and *LIG4b* (pLIG4b-R). V: vegetative cells. F: fragmented parental MAC. NM: cells with two visible new developing MACs. PA: post-autogamous cells with one MAC and surrounding fragments. Histograms on top show the progression of autogamy.
(TIF)

Figure S7 Molecular analysis of IES excision in *XRCC4*-silenced cells. Time-courses were performed with strain 51Δ*A* (same experiment as in Figure S4D). In each experiment, cells were transferred to RNAi medium at day 0 (D0) and starved to induce autogamy. V: vegetative cells. The T0-time point was arbitrarily chosen as the time when 50% of cells had a fragmented old MAC. F: cells with fragmented parental MAC. NM: cells with two new visible developing MACs. PA: post autogamous cells with only one new MAC and surrounding fragments. Only 3 IESs are shown: 3 = 51A4404 (77 bp), 5 = sm19-576 (66 bp) and 6 = 51G4404 (222 bp). Black lines: flanking MAC sequences, grey boxes: eliminated IES. A. Detection of excision junction for IES 51A4404. PCR around the IES allows the detection of a newly formed excision junction in the control but not in *XRCC4* silencing. Black arrowheads represent PCR primers. B. LMPCR analysis of double-strand breaks at IES boundaries. For each time-course, the progression of autogamy is displayed as a histogram of successive stages. Arrows indicate the position of DNA cleavage revealed by each molecular analysis.
(TIF)

Figure S8 Detection of single end-cleaved and linear excised IES molecules in *LIG4-*silenced cells. A & B. Detection of free 3′OH ends at the boundaries of IES #5 (sm19-576) during autogamy of strain 51Δ*A* silenced for *ND7* (Control) or *LIG4* expression. On each diagrammed molecule, the IES is represented by a grey box and its flanking DNA by a thick black line. The 3′ end tailed by the terminal transferase is indicated by a blue arrowhead. Poly(C)-tailed ends were amplified using primers sm19-5 and I (in A) or sm19-3 and I (in B, top). Ethidium bromide staining of 3% Nusieve agarose gels allowed only the detection of broken chromosome ends (A: expected size ∼211 bp; B: expected size ∼142 bp), while single-end cleaved IES molecules attached to their flanking DNA at their uncleaved end were not visible (expected sizes: ∼277 bp in A and ∼208 bp in B). Relevant size standards (in bp) are indicated on the left of each panel. In B (bottom), molecules carrying a DSB at the IES right end and still attached to their flanking DNA at the left end were revealed by ^33^P-labeled primer extension (using nested primer sm19-3-aval), followed by electrophoresis on high resolution denaturing gels. The identity of PCR and primer extension products was confirmed by sequencing of gel-purified DNA. C. Sequencing chromatograms of the poly(C)-tailed chromosome ends generated by DNA cleavage at the left boundary of IES #5 (“MAC left” molecules in B). D. LMPCR-mediated detection of excised linear forms of IES #6 (51G4404) during autogamy of strain 51 silenced for *ND7* (control) or *LIG4* expression. Following the ligation of linker (ATAC)J'/I', PCR amplification was carried out with primer I' only. 51G4404-specific products were revealed by Southern blot hybridization of 3% Nusieve agarose gels, using the ^32^P-labeled IES as a probe. The molecules to which the linker may be ligated are diagrammed below the pictures, with the linker and primer I' represented by a purple box and arrowhead, respectively.
(TIF)

Table S1 Oligonucleotides used in this study. Black arrowheads show the position of each primer relative to a particular IES (drawn as a double line). (-) represents MAC flanking sequences. Acc. Nb: ParameciumDB accession number. GenBank: when necessary, the GenBank accession number corresponding to MIC sequences is displayed. O: orientation relative to the transcriptional direction of each gene. [bp]: length of the expected PCR product. T°C: annealing temperature used for PCR amplification. PCR conditions were as follows: 2 min at 95°C, 25–37 cycles of 20–60 sec at 95°C, 20–60 sec at the appropriate annealing temperature, and 1 min at 72°C, followed by a final step of 3 min at 72°C. A. Primers in *LIG4a* gene. B. Primers in *XRCC4* gene. Two product sizes are indicated (with/without additional nucleotides on the 5′ end of each primer, in lower-case). The two annealing temperatures that were used successively for PCR amplification are shown. C. Primer in the 17S rDNA gene. D. Primers for MAC junction analysis. The two product lengths correspond to IES+ or IES- forms, respectively. E. Primers for circle junction analysis. For IES 51A2591, the two products lengths correspond to forms with or without the 28-bp internal IES, respectively. F. Primers used for LMPCR to detect free broken chromosome ends. Red arrowheads mark the position of the ligated linker and of primer PCRhaut, which hybridizes within the linker. PCRhaut is used to amplify ligation products, in combination with a second primer represented by a black arrowhead. Oligonucleotides used for primer extension (1 to 10 cycles) are drawn as white arrowheads. G. Primers used for LMPCR to detect free broken IES ends. Diagrams are as in F, with I' instead of PCRhaut. H. Primers used for the detection of free 3′OH ends at MAC ends. After a first elongation step with Anchor(G) (which hybridizes to the poly(C) tail added by TdT), the DNA is amplified by PCR using I and specific primers.
(PDF)

Text S1 Supplemental experimental procedures.
(PDF)

## References

[1] Keeney S, Neale MJ (2006). Initiation of meiotic recombination by formation of DNA double-strand breaks: mechanism and regulation.. Biochem Soc Trans.

[2] Soulas-Sprauel P, Rivera-Munoz P, Malivert L, Le Guyader G, Abramowski V (2007). V(D)J and immunoglobulin class switch recombinations: a paradigm to study the regulation of DNA end-joining.. Oncogene.

[3] Kapitonov VV, Jurka J (2005). RAG1 core and V(D)J recombination signal sequences were derived from Transib transposons.. PLoS Biol.

[4] Gellert M (2002). V(D)J recombination: RAG proteins, repair factors, and regulation.. Annu Rev Biochem.

[5] Lieber MR (2010). The Mechanism of Double-Strand DNA Break Repair by the Nonhomologous DNA End-Joining Pathway.. Annu Rev Biochem.

[6] Jahn CL, Klobutcher LA (2002). Genome remodeling in ciliated protozoa.. Annu Rev Microbiol.

[7] Yao MC, Duharcourt S, Chalker DL, Craig NL, Craigie R, Gellert M, Lambowitz AM (2002). Genome-wide rearrangements of DNA in ciliates.. Mobile DNA II.

[8] Prescott DM (1994). The DNA of ciliated protozoa.. Microbiol Rev.

[9] Le Mouel A, Butler A, Caron F, Meyer E (2003). Developmentally regulated chromosome fragmentation linked to imprecise elimination of repeated sequences in paramecia.. Eukaryot Cell.

[10] Bétermier M (2004). Large-scale genome remodelling by the developmentally programmed elimination of germ line sequences in the ciliate Paramecium.. Res Microbiol.

[11] Gratias A, Bétermier M (2003). Processing of double-strand breaks is involved in the precise excision of paramecium internal eliminated sequences.. Mol Cell Biol.

[12] Baudry C, Malinsky S, Restituito M, Kapusta A, Rosa S (2009). PiggyMac, a domesticated piggyBac transposase involved in programmed genome rearrangements in the ciliate Paramecium tetraurelia.. Genes Dev.

[13] Gratias A, Lepere G, Garnier O, Rosa S, Duharcourt S (2008). Developmentally programmed DNA splicing in Paramecium reveals short-distance crosstalk between DNA cleavage sites.. Nucleic Acids Res.

[14] Saveliev SV, Cox MM (1995). Transient DNA breaks associated with programmed genomic deletion events in conjugating cells of Tetrahymena thermophila.. Genes Dev.

[15] Williams K, Doak TG, Herrick G (1993). Developmental precise excision of Oxytricha trifallax telomere-bearing elements and formation of circles closed by a copy of the flanking target duplication.. Embo J.

[16] Bétermier M, Duharcourt S, Seitz H, Meyer E (2000). Timing of developmentally programmed excision and circularization of Paramecium internal eliminated sequences.. Mol Cell Biol.

[17] Aury JM, Jaillon O, Duret L, Noel B, Jubin C (2006). Global trends of whole-genome duplications revealed by the ciliate Paramecium tetraurelia.. Nature.

[18] Ellenberger T, Tomkinson AE (2008). Eukaryotic DNA ligases: structural and functional insights.. Annu Rev Biochem.

[19] Sinha KM, Hines JC, Downey N, Ray DS (2004). Mitochondrial DNA ligase in Crithidia fasciculata.. Proc Natl Acad Sci U S A.

[20] Downey N, Hines JC, Sinha KM, Ray DS (2005). Mitochondrial DNA ligases of Trypanosoma brucei.. Eukaryot Cell.

[21] McVey M, Lee SE (2008). MMEJ repair of double-strand breaks (director's cut): deleted sequences and alternative endings.. Trends Genet.

[22] Burton P, McBride DJ, Wilkes JM, Barry JD, McCulloch R (2007). Ku heterodimer-independent end joining in Trypanosoma brucei cell extracts relies upon sequence microhomology.. Eukaryot Cell.

[23] Critchlow SE, Bowater RP, Jackson SP (1997). Mammalian DNA double-strand break repair protein XRCC4 interacts with DNA ligase IV.. Curr Biol.

[24] Ahnesorg P, Smith P, Jackson SP (2006). XLF interacts with the XRCC4-DNA ligase IV complex to promote DNA nonhomologous end-joining.. Cell.

[25] Buck D, Malivert L, de Chasseval R, Barraud A, Fondaneche MC (2006). Cernunnos, a novel nonhomologous end-joining factor, is mutated in human immunodeficiency with microcephaly.. Cell.

[26] Frank KM, Sekiguchi JM, Seidl KJ, Swat W, Rathbun GA (1998). Late embryonic lethality and impaired V(D)J recombination in mice lacking DNA ligase IV.. Nature.

[27] Barnes DE, Stamp G, Rosewell I, Denzel A, Lindahl T (1998). Targeted disruption of the gene encoding DNA ligase IV leads to lethality in embryonic mice.. Curr Biol.

[28] Gao Y, Sun Y, Frank KM, Dikkes P, Fujiwara Y (1998). A critical role for DNA end-joining proteins in both lymphogenesis and neurogenesis.. Cell.

[29] Arnaiz O, Gout JF, Betermier M, Bouhouche K, Cohen J (2010). Gene expression in a paleopolyploid: a transcriptome resource for the ciliate Paramecium tetraurelia.. BMC Genomics.

[30] Berger JD (1973). Nuclear differentiation and nucleic acid synthesis in well-fed exconjugants of Paramecium aurelia.. Chromosoma.

[31] Kung C, Chang SY, Satow Y, Houten JV, Hansma H (1975). Genetic dissection of behavior in paramecium.. Science.

[32] Galvani A, Sperling L (2002). RNA interference by feeding in Paramecium.. Trends Genet.

[33] Berger JD (1973). Selective inhibition of DNA synthesis in macronuclear fragments in Paramecium aurelia exconjugants and its reversal during macronuclear regeneration.. Chromosoma.

[34] Matsuda A, Forney JD (2006). The SUMO pathway is developmentally regulated and required for programmed DNA elimination in Paramecium tetraurelia.. Eukaryot Cell.

[35] Berger JD (1974). Selective autolysis of nuclei as a source of DNA precursors in Paramecium aurelia exconjugants.. J Protozool.

[36] Duharcourt S, Lepere G, Meyer E (2009). Developmental genome rearrangements in ciliates: a natural genomic subtraction mediated by non-coding transcripts.. Trends Genet.

[37] Nowacki M, Landweber LF (2009). Epigenetic inheritance in ciliates.. Curr Opin Microbiol.

[38] Matsuda A, Shieh AW, Chalker DL, Forney JD (2010). The conjugation-specific Die5 protein is required for development of the somatic nucleus in both Paramecium and Tetrahymena.. Eukaryot Cell.

[39] Cheng CY, Vogt A, Mochizuki K, Yao MC (2010). A domesticated piggyBac transposase plays key roles in heterochromatin dynamics and DNA cleavage during programmed DNA deletion in Tetrahymena thermophila.. Mol Biol Cell.

[40] Nowacki M, Higgins BP, Maquilan GM, Swart EC, Doak TG (2009). A functional role for transposases in a large eukaryotic genome.. Science.

[41] Garnier O, Serrano V, Duharcourt S, Meyer E (2004). RNA-mediated programming of developmental genome rearrangements in Paramecium tetraurelia.. Mol Cell Biol.

[42] Budman J, Kim SA, Chu G (2007). Processing of DNA for nonhomologous end-joining is controlled by kinase activity and XRCC4/ligase IV.. J Biol Chem.

[43] Mitra R, Fain-Thornton J, Craig NL (2008). piggyBac can bypass DNA synthesis during cut and paste transposition.. Embo J.

[44] Guirouilh-Barbat J, Huck S, Bertrand P, Pirzio L, Desmaze C (2004). Impact of the KU80 pathway on NHEJ-induced genome rearrangements in mammalian cells.. Mol Cell.

[45] Arnaiz O, Cain S, Cohen J, Sperling L (2007). ParameciumDB: a community resource that integrates the Paramecium tetraurelia genome sequence with genetic data.. Nucleic Acids Res.

[46] Sibanda BL, Critchlow SE, Begun J, Pei XY, Jackson SP (2001). Crystal structure of an Xrcc4-DNA ligase IV complex.. Nat Struct Biol.

[47] Tamura K, Dudley J, Nei M, Kumar S (2007). MEGA4: Molecular Evolutionary Genetics Analysis (MEGA) software version 4.0.. Mol Biol Evol.

[48] Combet C, Blanchet C, Geourjon C, Deleage G (2000). NPS@: network protein sequence analysis.. Trends Biochem Sci.

[49] Timmons L, Fire A (1998). Specific interference by ingested dsRNA.. Nature.

[50] Edgar RC (2004). MUSCLE: multiple sequence alignment with high accuracy and high throughput.. Nucleic Acids Res.

[51] Gong C, Bongiorno P, Martins A, Stephanou NC, Zhu H (2005). Mechanism of nonhomologous end-joining in mycobacteria: a low-fidelity repair system driven by Ku, ligase D and ligase C.. Nat Struct Mol Biol.

[52] Zhu H, Shuman S (2007). Characterization of Agrobacterium tumefaciens DNA ligases C and D.. Nucleic Acids Res.

[53] Gaboriaud C, Bissery V, Benchetrit T, Mornon JP (1987). Hydrophobic cluster analysis: an efficient new way to compare and analyse amino acid sequences.. FEBS Lett.

[54] Callebaut I, Courvalin JC, Worman HJ, Mornon JP (1997). Hydrophobic cluster analysis reveals a third chromodomain in the Tetrahymena Pdd1p protein of the chromo superfamily.. Biochem Biophys Res Commun.

[55] Eudes R, Le Tuan K, Delettre J, Mornon JP, Callebaut I (2007). A generalized analysis of hydrophobic and loop clusters within globular protein sequences.. BMC Struct Biol.

[56] Callebaut I, Malivert L, Fischer A, Mornon JP, Revy P (2006). Cernunnos interacts with the XRCC4 x DNA-ligase IV complex and is homologous to the yeast nonhomologous end-joining factor Nej1.. J Biol Chem.

