# Supplementary figures and images for: Highly Precise and Developmentally Programmed Genome Assembly in Paramecium Requires Ligase IV–Dependent End Joining
Source: PLoS Genet. 2011 Apr 14;7(4):e1002049. doi: 10.1371/journal.pgen.1002049 (PMC3077386; doi:10.1371/journal.pgen.1002049)

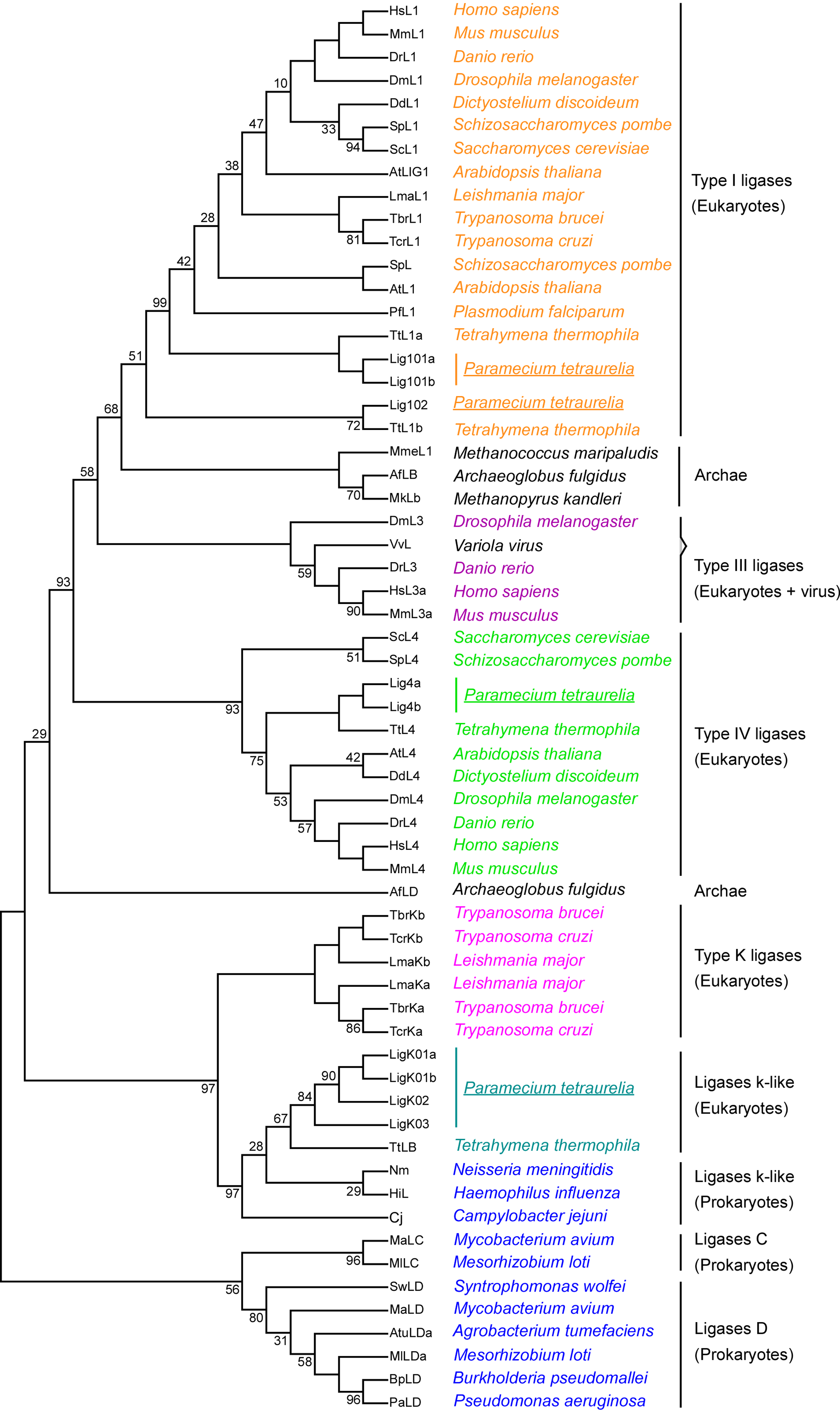

Supplement: Figure S1 — Phylogenetic tree of ATP-dependent DNA ligases. Amino-acid sequences of 61 ATP-dependent DNA Ligases were aligned using the MUSCLE algorithm [50]. Non-informative regions were removed manually from the alignment. The phylogenetic tree was generated by neighbor-joining using MEGA 4, with the following parameters: bootstrap 1000, pairwise deletion of gaps, equal input model, heterogeneous pattern among lineages, gamma distributed rates among site (with a gamma shape parameter = 1.7, as estimated by the means of gamma parameters calculated with the PhyML algorithm at http://www.hiv.lanl.gov). Bootstrap values are not shown when equal to 100. Prokaryotic NAD+-dependent replicative ligases (LigA) are not included. Some bacteria possess ATP-dependent DNA ligases (Ligases C or D) involved in DNA repair [51], [52]. Some also encode ATP-dependent type K Ligase homologues: 3 randomly selected bacterial LigK are represented. ParameciumDB accession numbers of Paramecium genes are provided in the legend to Figure 1. TtL1a = XP_001022972.1 on GenBank. For all other proteins, Uniprot accession numbers are: TtL1b = Q24FD9, TtL4 = Q23RI5, TtlK = Q233G4, LmaL1 = Q4Q6U5, LmaKa = Q4Q960, LmaKb = Q4Q959, TbrL1 = Q587E4, TbrKa = Q6V9I8, TbrKb = Q56AN9, TcrL1a = Q4DX91, TcrKa = Q4DMH8, TcrKb = Q4DMH7, AtL1 = Q9C9M5, AtLIG1 = Q42572, AtL4 = Q9LL84, HsL4a = P49917, MmL1 = P37913, MmL4 = Q8BTF7, SpL = Q9C1W9, SpL4 = O74833, MkLb = Q8TWN3, MmeL1 = A6VFQ9, VvL = P33798, AfLD = O28549, AfLB = O29632, AtuLDa = A9CLR5, Cj = Q5HSC4, BpLD = Q63I59, HiL = P44121, MaLC = Q744K0, MaLD = Q742F5, MlLC = Q98NY5, MlLDa = Q98DP8, Nm = C6S4M8, PaLD = Q9I1X7, SwLD = Q0AXX1. (TIF) [file pgen.1002049.s001.tif]

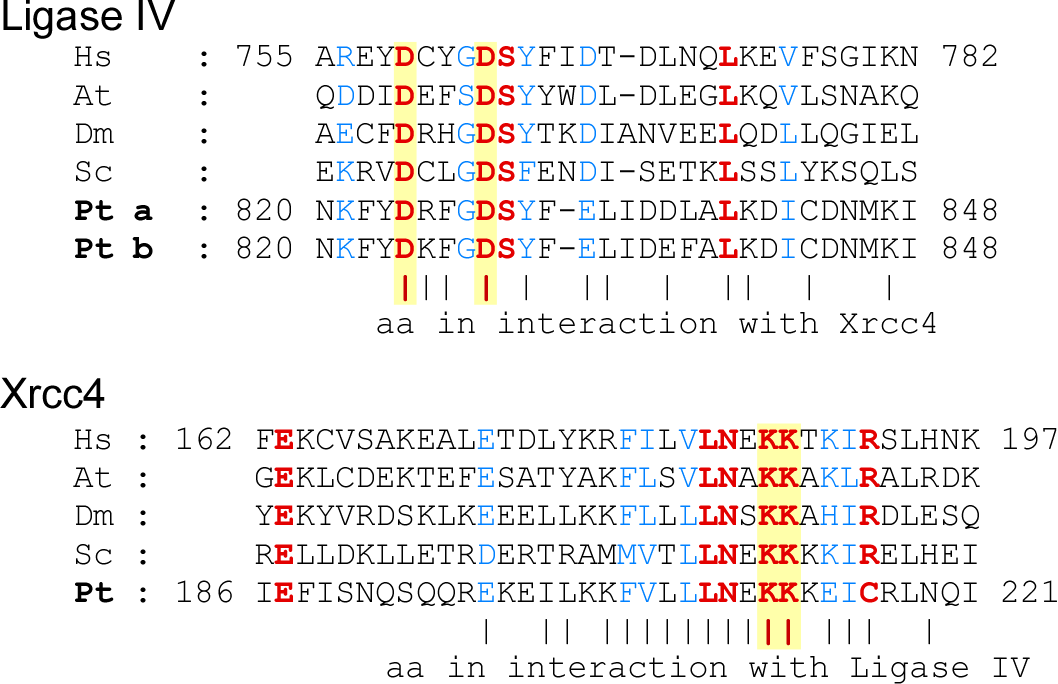

Supplement: Figure S2 — Conservation of the amino acids responsible for the Xrcc4–DNA ligase IV interaction. A multiple sequence alignment of the Lig4p-interacting region of Xrcc4p homologs from different organisms is displayed in the top panel. The linker regions connecting the BRCT domains of Lig4p homologs and involved in the interaction with Xrcc4p, are aligned in the bottom panel. Amino acids (aa) involved in the interaction between human ligase IV and Xrcc4p are shown (|). Strongly conserved residues are in cyan, identical in bold and red, and essential residues are highlighted in yellow [46]. Hs: Homo sapiens, At: Arabidopsis thaliana, Dm: Drosophila melanogaster, Sc: Saccharomyces cerevisiae, Pt: Paramecium tetraurelia. (TIF) [file pgen.1002049.s002.tif]

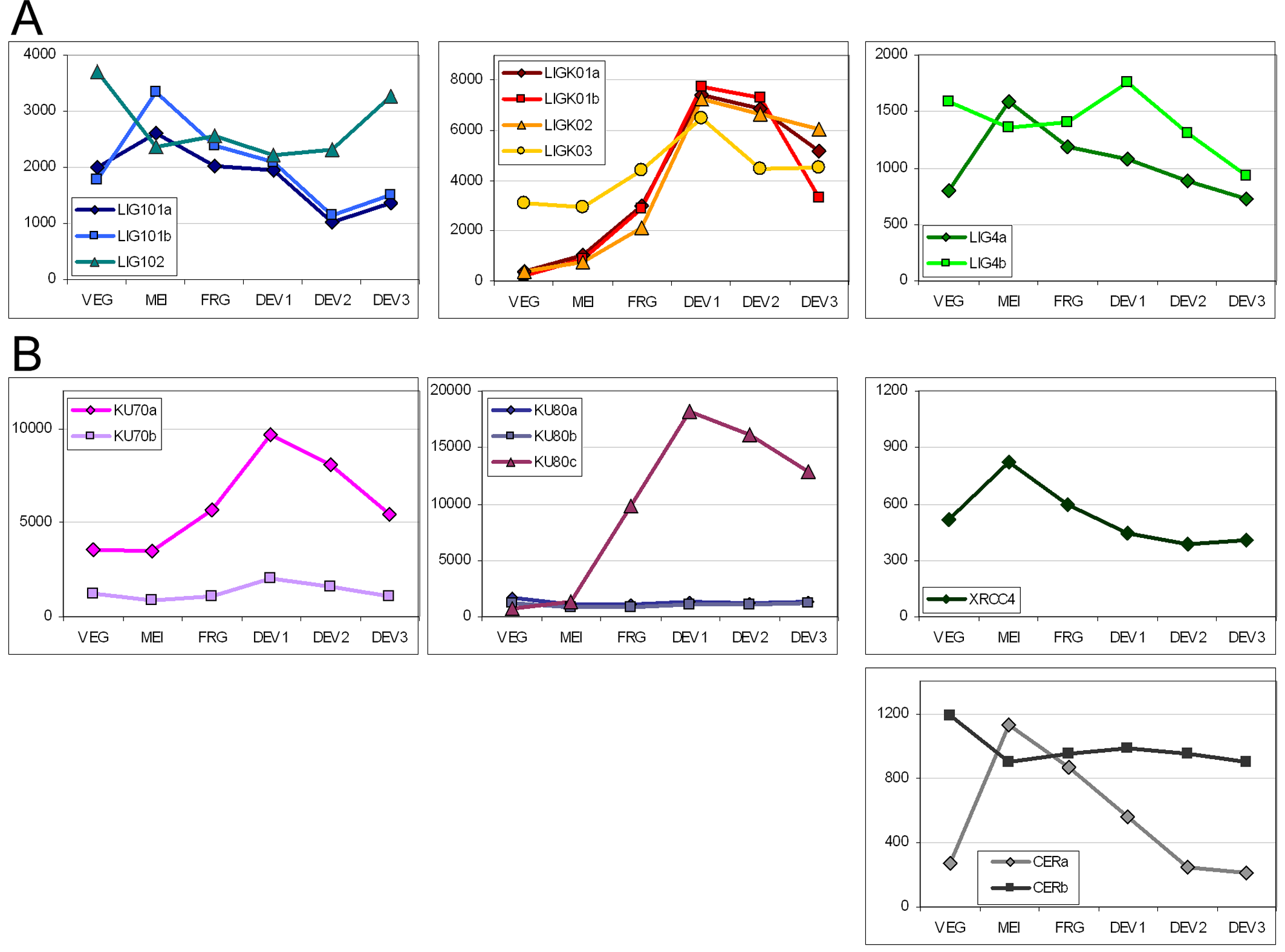

Supplement: Figure S3 — Expression of P. tetraurelia DNA ligase and NHEJ genes during autogamy. NimbleGen whole-genome microarrays carry 6 oligonucleotide probes per gene: each slide was hybridized with a cDNA sample and the median of the six signals was calculated ([29], data available in ParameciumDB). Because all oligonucleotide probes present on the slides do not have the same Tm, microarrays do not allow comparing absolute transcript levels for different genes and only provide information on the relative variations of expression for each individual gene during autogamy. Following clusterization of slides, autogamy stages were defined as follows: VEG, vegetative cells; MEI, MIC meiosis; FRG, fragmented old MAC and no detectable new MAC following DAPI staining; DEV, visible developing new MACs (1, 2, 3 refer to three successive stages). Expression profiles are drawn from the mean values obtained for each stage from 4 independent time-course experiments. A. Paramecium ATP-dependent DNA ligases. For the particular case of LIG4b, automatic annotation of the draft MAC P. tetraurelia genome has split the gene into two open reading frames (GSPATG00022021001 and GSPATG00022020001). Thus, two sets of 6 probes contribute to the LIG4b signal and the curve represents the median of all 12 probes for each stage. This analysis reveals that two out of three LIG1 genes are induced early during sexual processes, as expected for a postulated role in DNA replication, during meiosis or MAC development. The four LIGK are strongly upregulated at later stages during MAC development: future work should provide more insight into their function. B. Core NHEJ genes present in the P. tetraurelia genome. XRCC4 accession number in ParameciumDB can be found in the legend to Figure 4A. Other ParameciumDB accession numbers are: GSPATG00006445001 for KU70a, GSPATG00009747001 for KU70b, GSPATG00034664001 for KU80a, GSPATG00035446001 for KU80b and GSPATG00030095001 for KU80c. Putative CERNUNNOS (CER) genes are also repres [file pgen.1002049.s003.tif]

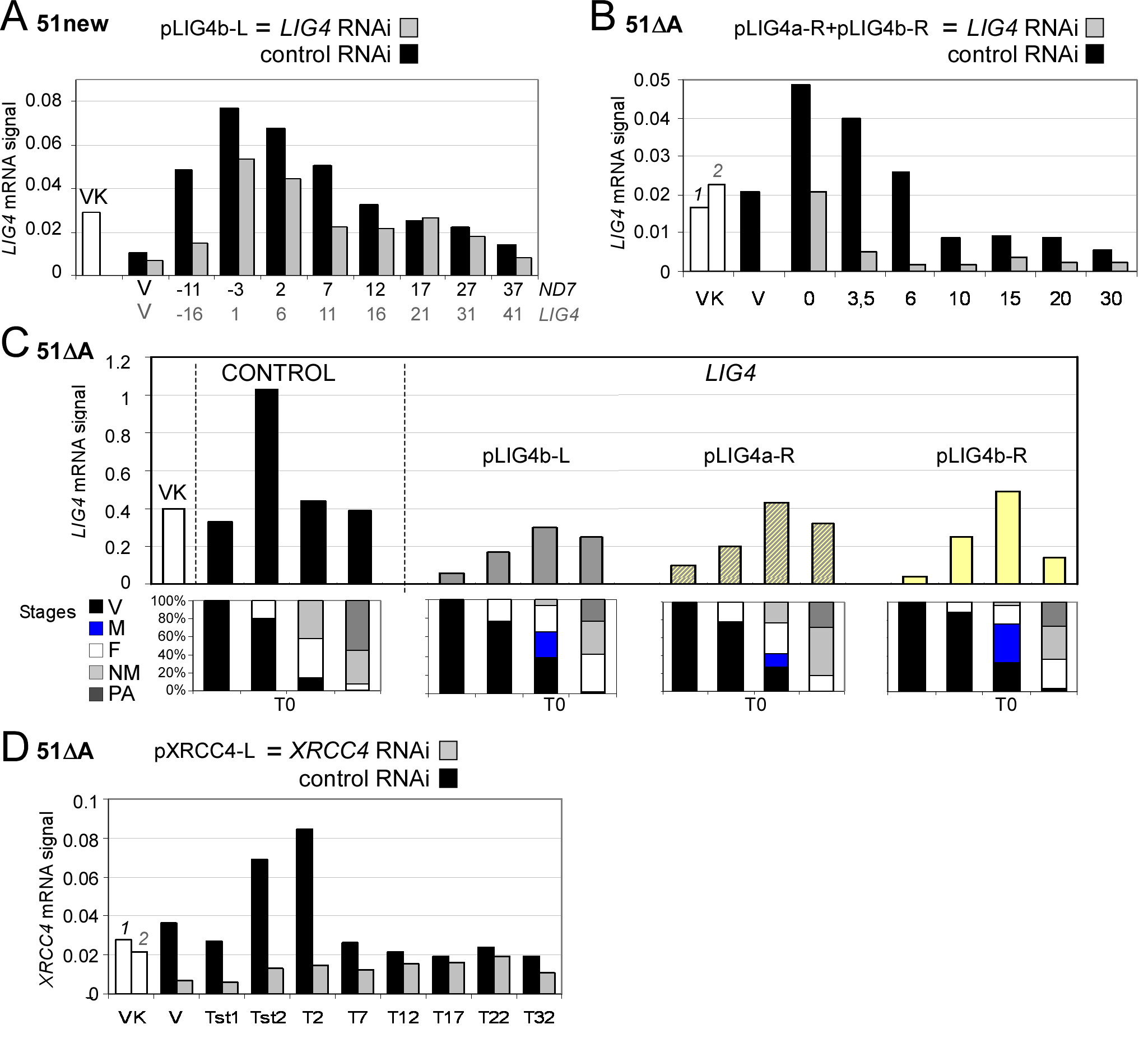

Supplement: Figure S4 — Quantification of LIG4 and XRCC4 mRNA levels in silenced cells. Northern blots of total RNA extracted during different autogamy time-courses were probed with LIG4 (A, B & C) or XRCC4 (D) probes. All values were normalized with 17S rDNA signal for quantification. VK corresponds to vegetative cells grown in standard bacteria before transfer to silencing medium. The t = 0 hrs time-point was set as the time when 50% of cells had a fragmented old MAC. Times are in hours. A. RNAi was carried out on strain 51 (same experiment as in Figure 6), using pLIG4b-L (Figure 1) to induce the production of LIG4 dsRNA. Total RNA samples extracted from both LIG4- and ND7-silenced cells were blotted onto a single membrane and hybridized to a LIG4 probe. Time-points of ND7 and LIG4 RNAi experiments are displayed separately. B. RNAi against both LIG4 genes was performed on strain 51ΔA (same experiment as in Figure 7 and Figure S6), using pLIG4a-R and pLIG4b-R (Figure 4A) for dsRNA production. Samples from LIG4 and ND7 silencing experiments were transferred separately to two different northern blots and the VK sample was loaded in duplicate on each membrane (1: LIG4 blot; 2: ND7 blot). The two blots were hybridized together with a LIG4 probe. C. Three different dsRNA-producing constructs were tested separately to induce RNAi against LIG4 genes in strain 51ΔA. All samples were loaded on the same blot and hybridized with a LIG4 probe. Silencing using pLIG4a-R or pLIG4b-R was found to be as efficient as with pLIG4b-L, based on northern blot hybridization and on high lethality rates in the progeny of silenced cells. Autogamy stages are displayed below each diagram. V: vegetative cells; M: MIC meiosis; F: fragmented old MAC; NM: two visible new developing MACs; PA: post-autogamous cells with one MAC and surrounding fragments. D. RNAi against XRCC4 was applied to strain 51ΔA using plasmid pXRCC4-L (Figure 4A). RNA samples from XRCC4- and ND7-silenced cells (same experiment as in Figure S7) were [file pgen.1002049.s004.tif]

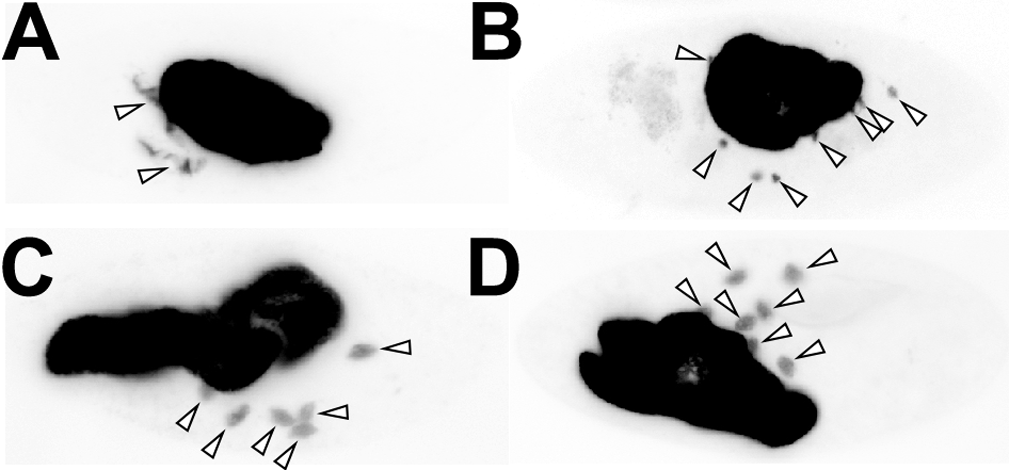

Supplement: Figure S5 — Normal progression of meiosis during the silencing of LIG4 or XRCC4. DAPI staining of single cells fixed during autogamy in the macronuclear variant 51ΔA. Cells were not treated with RNase prior to staining. During LIG4 silencing (A, C & D), starved cells were fixed 1 day following transfer to RNAi medium. For XRCC4 silencing (B), the sample was treated at day 2. White arrows: micronuclear meiotic products. The heavily stained nucleus in each panel corresponds to the MAC. A. First meiotic division. B, C and D. After meiosis II. The eight haploid mics are sometimes not all visible, when they are not in the same focus. (TIF) [file pgen.1002049.s005.tif]

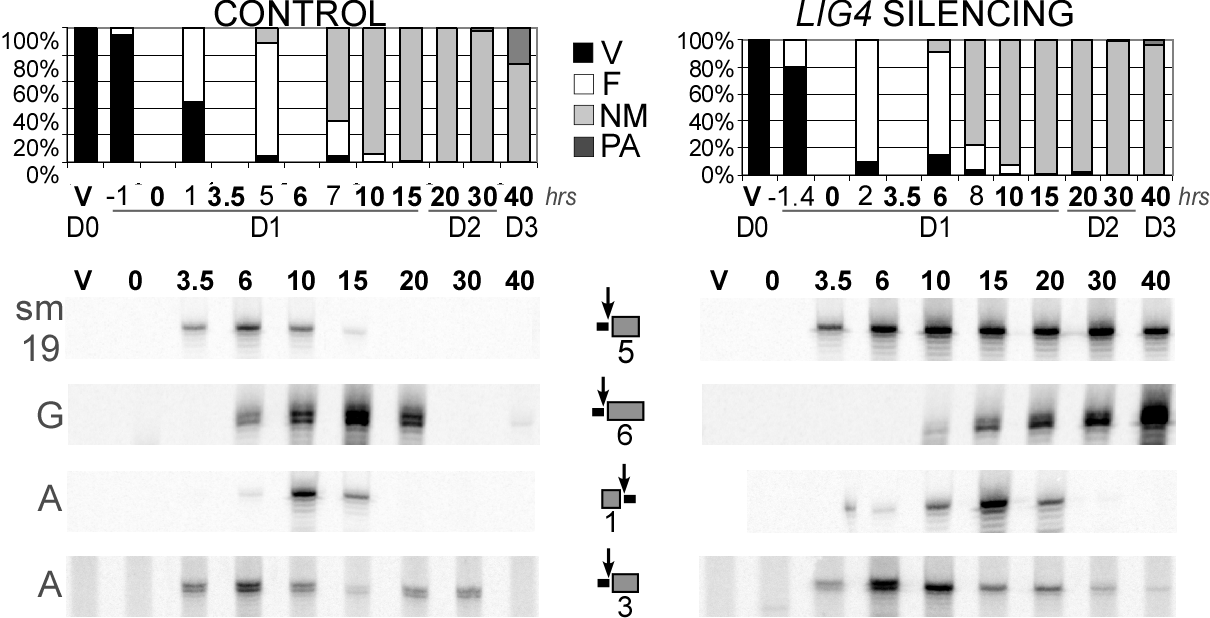

Supplement: Figure S6 — LMPCR detection of DSBs during autogamy of the macronuclear variant 51ΔA silenced for LIG4. Four IESs are presented: 1 = 51A1835 (28 bp), 3 = 51A4404 (77 bp) from surface antigen A gene; 5 = sm19-576 (66 bp) from SM19 tubulin gene and 6 = 51G4404 (222 bp) from surface antigen G gene. Gene names are indicated on the left of panels A and B. MAC flanking sequences are drawn as black lines and IESs as grey boxes. Vertical arrows indicate the position of DNA cleavage in each experiment. In all time-courses, cells were transferred to RNAi medium at day 0 (D0). The T0-time point is the time when 50% of cells have a fragmented old MAC. RNAi against LIG4 was obtained using mixed bacterial cultures producing dsRNA from LIG4a (pLIG4a-R) and LIG4b (pLIG4b-R). V: vegetative cells. F: fragmented parental MAC. NM: cells with two visible new developing MACs. PA: post-autogamous cells with one MAC and surrounding fragments. Histograms on top show the progression of autogamy. (TIF) [file pgen.1002049.s006.tif]

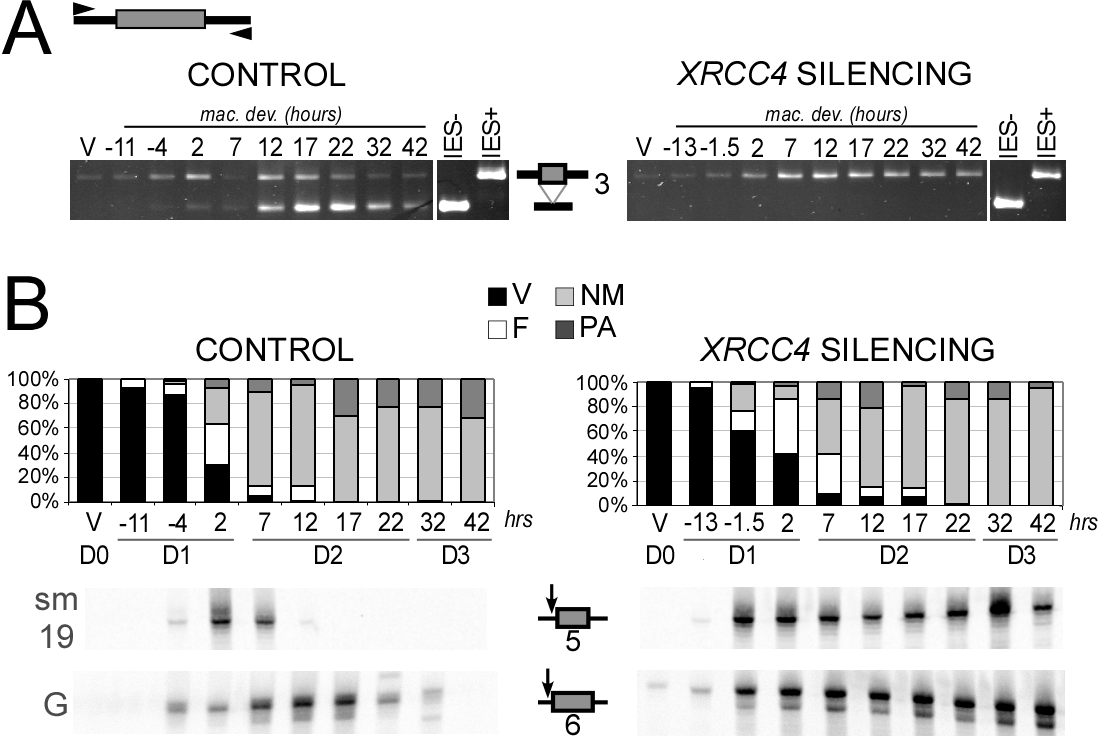

Supplement: Figure S7 — Molecular analysis of IES excision in XRCC4-silenced cells. Time-courses were performed with strain 51ΔA (same experiment as in Figure S4D). In each experiment, cells were transferred to RNAi medium at day 0 (D0) and starved to induce autogamy. V: vegetative cells. The T0-time point was arbitrarily chosen as the time when 50% of cells had a fragmented old MAC. F: cells with fragmented parental MAC. NM: cells with two new visible developing MACs. PA: post autogamous cells with only one new MAC and surrounding fragments. Only 3 IESs are shown: 3 = 51A4404 (77 bp), 5 = sm19-576 (66 bp) and 6 = 51G4404 (222 bp). Black lines: flanking MAC sequences, grey boxes: eliminated IES. A. Detection of excision junction for IES 51A4404. PCR around the IES allows the detection of a newly formed excision junction in the control but not in XRCC4 silencing. Black arrowheads represent PCR primers. B. LMPCR analysis of double-strand breaks at IES boundaries. For each time-course, the progression of autogamy is displayed as a histogram of successive stages. Arrows indicate the position of DNA cleavage revealed by each molecular analysis. (TIF) [file pgen.1002049.s007.tif]

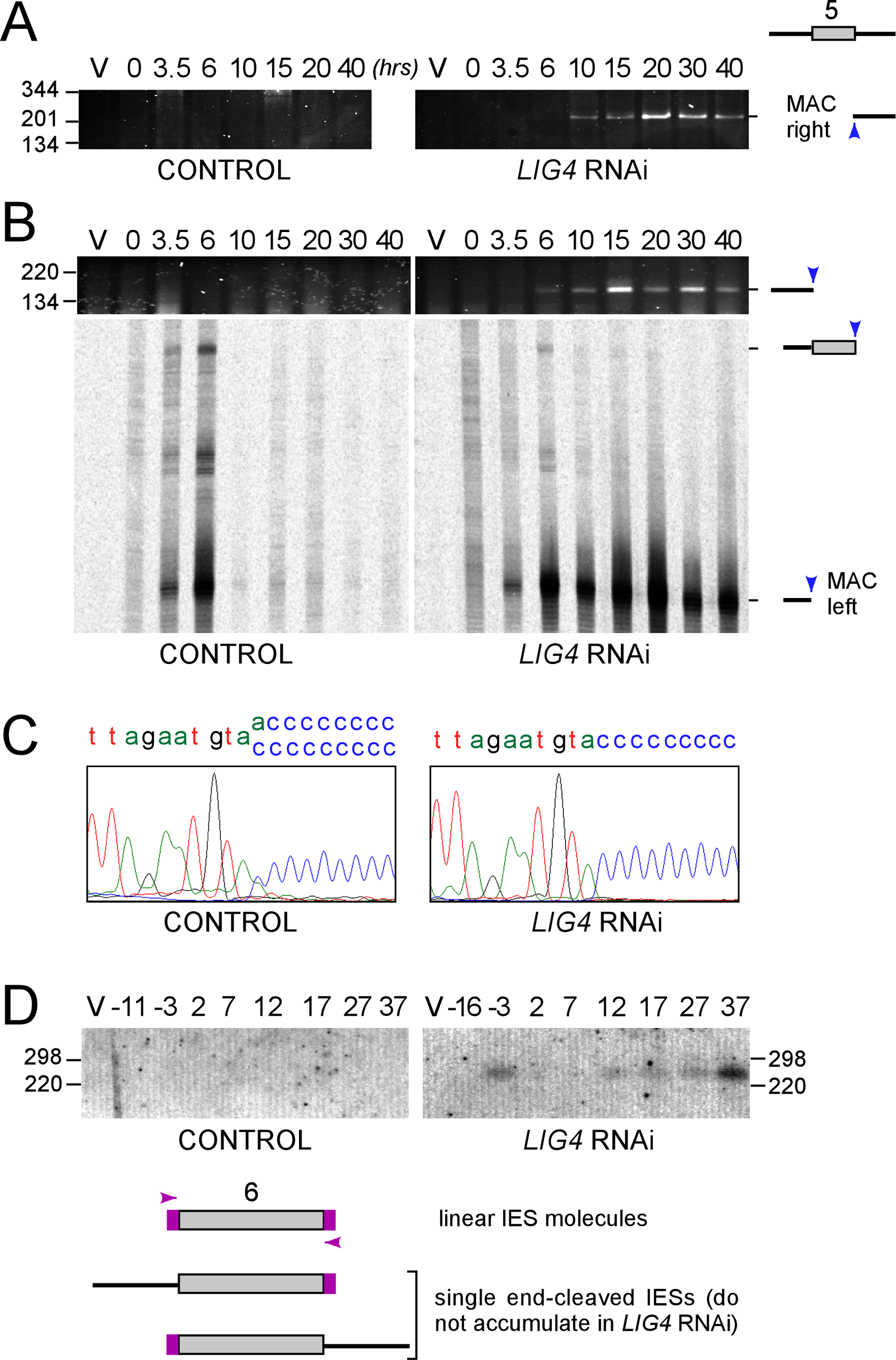

Supplement: Figure S8 — Detection of single end-cleaved and linear excised IES molecules in LIG4-silenced cells. A & B. Detection of free 3′OH ends at the boundaries of IES #5 (sm19-576) during autogamy of strain 51ΔA silenced for ND7 (Control) or LIG4 expression. On each diagrammed molecule, the IES is represented by a grey box and its flanking DNA by a thick black line. The 3′ end tailed by the terminal transferase is indicated by a blue arrowhead. Poly(C)-tailed ends were amplified using primers sm19-5 and I (in A) or sm19-3 and I (in B, top). Ethidium bromide staining of 3% Nusieve agarose gels allowed only the detection of broken chromosome ends (A: expected size ∼211 bp; B: expected size ∼142 bp), while single-end cleaved IES molecules attached to their flanking DNA at their uncleaved end were not visible (expected sizes: ∼277 bp in A and ∼208 bp in B). Relevant size standards (in bp) are indicated on the left of each panel. In B (bottom), molecules carrying a DSB at the IES right end and still attached to their flanking DNA at the left end were revealed by 33P-labeled primer extension (using nested primer sm19-3-aval), followed by electrophoresis on high resolution denaturing gels. The identity of PCR and primer extension products was confirmed by sequencing of gel-purified DNA. C. Sequencing chromatograms of the poly(C)-tailed chromosome ends generated by DNA cleavage at the left boundary of IES #5 (“MAC left” molecules in B). D. LMPCR-mediated detection of excised linear forms of IES #6 (51G4404) during autogamy of strain 51 silenced for ND7 (control) or LIG4 expression. Following the ligation of linker (ATAC)J'/I', PCR amplification was carried out with primer I' only. 51G4404-specific products were revealed by Southern blot hybridization of 3% Nusieve agarose gels, using the 32P-labeled IES as a probe. The molecules to which the linker may be ligated are diagrammed below the pictures, with the linker and primer I' represented by a purple box and arrowhead, respectively. (TIF) [file pgen.1002049.s008.tif]
