# Supplementary material for: Highly Precise and Developmentally Programmed Genome Assembly in Paramecium Requires Ligase IV–Dependent End Joining
Source: PLoS Genet. 2011 Apr 14;7(4):e1002049. doi: 10.1371/journal.pgen.1002049 (PMC3077386; doi:10.1371/journal.pgen.1002049)
Supplement: Table S1 — Oligonucleotides used in this study. Black arrowheads show the position of each primer relative to a particular IES (drawn as a double line). (-) represents MAC flanking sequences. Acc. Nb: ParameciumDB accession number. GenBank: when necessary, the GenBank accession number corresponding to MIC sequences is displayed. O: orientation relative to the transcriptional direction of each gene. [bp]: length of the expected PCR product. T°C: annealing temperature used for PCR amplification. PCR conditions were as follows: 2 min at 95°C, 25–37 cycles of 20–60 sec at 95°C, 20–60 sec at the appropriate annealing temperature, and 1 min at 72°C, followed by a final step of 3 min at 72°C. A. Primers in LIG4a gene. B. Primers in XRCC4 gene. Two product sizes are indicated (with/without additional nucleotides on the 5′ end of each primer, in lower-case). The two annealing temperatures that were used successively for PCR amplification are shown. C. Primer in the 17S rDNA gene. D. Primers for MAC junction analysis. The two product lengths correspond to IES+ or IES- forms, respectively. E. Primers for circle junction analysis. For IES 51A2591, the two products lengths correspond to forms with or without the 28-bp internal IES, respectively. F. Primers used for LMPCR to detect free broken chromosome ends. Red arrowheads mark the position of the ligated linker and of primer PCRhaut, which hybridizes within the linker. PCRhaut is used to amplify ligation products, in combination with a second primer represented by a black arrowhead. Oligonucleotides used for primer extension (1 to 10 cycles) are drawn as white arrowheads. G. Primers used for LMPCR to detect free broken IES ends. Diagrams are as in F, with I' instead of PCRhaut. H. Primers used for the detection of free 3′OH ends at MAC ends. After a first elongation step with Anchor(G) (which hybridizes to the poly(C) tail added by TdT), the DNA is amplified by PCR using I and specific primers. (PDF) [file pgen.1002049.s009.pdf]

A. Primers in *LIG4a* gene

Oligo name	Sequence (5'-3')	Note	Acc. Nb	O.	[bp]	T°C
ligIV-1_ATGup	ATGCAGCAGGAGAATTTTATAGACGAAGAG	Used for *LIG4a* probe	PTETG5400008001	+	510	62
ligIV-1_500R	ATTATGCACAACCATGGGGAAATCACCAAC			-		

B. Primers in *XRCC4* gene

Oligo name	Sequence (5'-3')	Note	Acc. Nb	O.	[bp]	T°C
XRCC4_302_BglII_U	atgcagatctAGGAAATAATGTATAAGAGTTTAGAAG	Used for *XRCC4* probe,	GSPATG00029540001	+	322/	53/
XRCC4_302_PstI_R	atgcctgcagTTACTCTTCATATTGTTTCTTAACTGG	in *XRCC4* silencing		-	302	61.5
XRCC4-496_U	GAATAAAAAAGAAATTAAACAAGATGGCG	Used for probe in wild type,	GSPATG00029540001	+	496	56
XRCC4-496_R	AAATCTCCTTGCAAACCCTGTAAAAATGG	and as RNAi fragment		-		

C. Primer in 17S rDNA

Oligo name	Sequence (5'-3')	Note
17Sext_NcoI	ACCCGTGACTGCCATGGTAGTCCAATACA	Used as oligo probe on Northern Blot

D. MAC junction analysis

IES	Oligo name	Sequence (5'-3')	Diagram	GenBank	O.	[bp]	T°C
A1835	51A1835-5'	TAATGTATTGATAAGGCTTGCTCTACAGCC	– ► – === – – –	L26124	+	240/211	60
	51A1835-3'(4)	AGACAAGTAGGGAATCCACTTCTAGTAATC	– – – === – ◄ –		-		
A2591	51A2591-5'	ACACCAAGCGAAACATGCACAGTCG	– ► – === – – –	L26124	+	476/106	54
	51A2591-3'	TTTTATGGCATTAAGCTTGTGTCAT	– – – === – ◄ –		-		
A4404	51A4404-5'	TAAATGTTCAGCTTACAACGCAGCT	– ► – === – – –	L26124	+	263/189	56
	51A4404-3'(2)	CCAGTTATTGAACTGCAACTTACTGCAGTG	– – – === – ◄ –		-		

E. Circle junction analysis

IES	Oligo name	Sequence (5'-3')	Diagram	GenBank	O.	[bp]	T°C
A2591	51A2591-09	CAATATTATACATCTAGAACTTATAGTTAG	– – – – = ◄ = – – – –	L26124	+	370/342	55.5
	51A2591-01	AGATTTATATCTTTTTTCTCAAATTCAGC	– – – – = ► = – – – –		-		
A4578	51A4578-2	TGGTTGTTAGTCTCAAAGAATTCTAAAGAC	– – – – = ◄ = – – – –	L26124	+	408	55.8
	51A4578-7	AAGAAATTTTATTGTAAATATATTTTCAGC	– – – – = ► = – – – –		-		
G4404	51G07	TTTTGAAATATTTTCAAGTTTTTGGACTAC	– – – – = ◄ = – – – –	AJ010441	+	222	54
	51G08	ACAATATATATTTACTTGATAATATTTTCC	– – – – = ► = – – – –		-		

F. LMPCR - Detection of free broken chromosome ends

IES	Oligo name	Sequence (5'-3')	Diagram	GenBank	O.	[bp]	T°C
	PCRhaut (▶)	GAATTCGGATCCGCTCGGACCGTGGC	used with specific primer ▶ to amplify the ligated product				
A1835	51A1835-3'(3)	GTAGTACAAGATTTTTCGACACAAGTTGAG	– – – === ▶ – – ◀ –	L26124	-	225	60
	51A1835-3'(4)	AGACAAGTAGGGAATCCACTTCTAGTAATC	– – – === – – ◁ – –		-	180	62
	51A1835-5'(3)	GGTTGCGTAACACTTCCTCTTAAATGTGAG	– ▶ – – ◀ === – – –		+	206	63
	51A1835-5'(4)	GAAGTCTAATGGATAACCTTGTGGATGGAC	– – ▷ – – === – – –		+	147	63
A2591	51A2591-16	AATTGTAAATTGACTTCAGCAAATAAAAAA	– ▶ – – ◀ === – – –	L26124	+	218	60
	51A2591-17	ATGTGTTTGGACTGGATTGGCATGTAGAAG	– – ▷ – – === – – –		+	189	63
A4404	51A4404-5'(2)	TGGAATAGTGCTGCATCACCAGCTGCTTGC	– ▶ – – ◀ === – – –	L26124	+	177	63
	51A4404-5'(3)	ACCAGCTGCTTGCATTCAAATATCCACAGT	– – ▷ – – === – – –		+	160	63
G4404	51G18	ACTGTTGCTACACATTGTGCATATGTTACT	– ▶ – – ◀ === – – –	AJ010441	+	135	60
	51G13	TGCATATGTTACTGGAACTGGATTGGTAGC	– – ▷ – – === – – –		+	118	63
sm19-576	sm19-2	AATTAAGCAAGAAAAGAAATAGAAAAAACC	– ▶ – – ◀ === – – –	AJ272425	+	188	54
	sm19-3	CTACAATAATGAGTCTAGCTGGTGGCACTG	– – ▷ – – === – – –	(mac sequence)	+	139	63

G. LMPCR - Detection of free broken IES ends

IES	Oligo name	Sequence (5'-3')	Diagram	GenBank	O.	[bp]	T°C
	I' (▶)	GCTCGGACCGTGGCTAGCATTAGTC	used with specific primer ▶ to amplify the ligated product				
G4404	51G11	GGACTACTTTTGAAATTGAATTATAACAAAGGC	– – ▶ === ◀ = – – –	AJ010441	-	154	60
	51G10	AAAGGCTAATTTGGATGAATGAGCATTAAATC	– – – == ◁ == – – –		-	127	60

H. Detection of free broken 3'OH ends

IES	Oligo name	Sequence (5'-3')	Diagrams and Notes	GenBank	O.	T°C
	I' (▶)	GCTCGGACCGTGGCTAGCATTAGTC				
	Anchor(G)	GCTCGGACCGTGGCTAGCATTAGTGAGTGGGGGGGGGGGGGGGG				
G4404	51G18	ACTGTTGCTACACATTGTGCATATGTTACT	–▶––◀ === –––	AJ010441	+	60
	51G13	TGCATATGTTACTGGAACTGGATTGGTAGC	––▷–– === ––– (primer extension and sequencing)		+	63
sm19-576	sm19-3	CTACAATAATGAGTCTAGCTGGTGGCACTG	–▶––◀ === ––– –▶–– === ◀–––	AJ272425 (mac)	-	64
	sm19-5	ATCCACACTTTTATCGATTTGCTTTGATCC	––– === ▶––◀– –––▶ === ––◀–		-	60
	sm19-3-aval	GTGGCACTGGATCCGGTCTAGGTAGTAG	––– === ––◁–– (primer extension and sequencing)		-	64
	sm19-6	ACTTAACATTTAATATATCCTGTCATTCTC	to sequence sm19-5/I PCR productss		-	
