# Supplementary material for: Highly Precise and Developmentally Programmed Genome Assembly in Paramecium Requires Ligase IV–Dependent End Joining
Source: PLoS Genet. 2011 Apr 14;7(4):e1002049. doi: 10.1371/journal.pgen.1002049 (PMC3077386; doi:10.1371/journal.pgen.1002049)
Supplement: Text S1 — Supplemental experimental procedures. (PDF) [file pgen.1002049.s010.pdf]

Supplemental experimental procedures

Analysis of GFP fusions

Approximately 2 pL of circular pLIG401GC (3 μg/μL) and pRB35 (1 μg/mL) in distilled water were injected into the macronucleus of vegetative a3093 cells under an inverted light microscope. For fixation of GFP-expressing cells, about 10-20 cells were concentrated by removing surrounding medium to less than 10 μL, then quickly mixed with 4% paraformaldehyde in phosphate-buffered saline, pH 7.4 (PBS). Cells were washed twice with PBS, treated with 0.1 mg/ml RNaseA, then mounted and counterstained by VECTASHIELD with DAPI (Vector Laboratories, Burlingame, CA). For quantitative observations, cells were flattened by gentle pressure on coverslips, with $\sim$5 μL rice glue cushions at the four corners. Fluorescence microscope (Olympus, Japan) images were obtained using a CCD camera (Spot, France) with fixed gain and exposure time.

Quantitative measurements of GFP signals were made with ImageJ (National Institutes of Health) and total fluorescence was calculated as follows:

$$\text{Total fluorescence (count)} = (I - B) \times A$$

where I is the mean pixel intensity measured for each object (mating pairs or individual starved or exconjugant cells), B is the background pixel intensity and A (in pixels) is the area of the object (measured under visible light).

RNAi plasmids

All RNAi plasmids are derivatives of vector L4440 (Timmons and Fire, 1998) and carry a target gene fragment between two convergent T7 promoters. Cloned fragments were as follows: nt 521-1774 from *LIG4b* for pLIG4b-L, 1824-2596 from *LIG4a* for pLIG4a-R, 1824-2596 from *LIG4b* for pLIG4b-R and 3-498 from *XRCC4* for pXRCC4-R (coordinates in nucleotides from the ATG start codon of each gene). Control RNAi plasmids were p0ND7c (Garnier et al., 2004) and pICL7a (Gogendeau et al., 2008), which target *ND7* and *ICL7a* non essential genes, respectively. RNA interference was achieved as described (Galvani and Sperling, 2002), by feeding *Paramecium* cells with *Escherichia coli* HT115 bacteria transformed with each plasmid and induced for the production of double strand RNA corresponding to each RNAi insert.

Quantification of nuclear DNA content

During conjugation, 20 cells from each time-point were fixed and treated with RNase as described above and nuclear DNA was stained by VECTASHIELD with propidium iodide.

For DNA quantification, cells were flattened as described above and observed with a standard fluorescent microscope. To compensate for staining differences between slides, the fluorescence signal from new MACs was normalized relative to surrounding old MAC fragments (in which DNA has stopped to replicate but is not degraded under the experimental conditions used). DNA content in the new developing MAC (D) was calculated as follows:

$$D = \frac{(N-B)}{(O-B)} \times A$$

where N is the mean pixel intensity measured in the new MAC, B is background intensity, O is an average of fluorescence intensity in three old MAC fragments and A is the new macronucleus two-dimensional area. Since cells were well flattened, two-dimensional measurement was sufficient for this case. For each time-point, 8 to 15 cells were used for measurement.

References

Garnier, O., Serrano, V., Duharcourt, S., and Meyer, E. (**2004**). "RNA-mediated programming of developmental genome rearrangements in Paramecium tetraurelia." *Mol Cell Biol*, 24(17): 7370–9.

Gogendeau, D., Klotz, C., Arnaiz, O., Malinowska, A., Dadlez, M., de Loubresse, N. G., Ruiz, F., Koll, F., and Beisson, J. (**2008**). "Functional diversification of centrins and cell morphological complexity." *J Cell Sci*, 121(Pt 1): 65–74.

Timmons, L. and Fire, A. (**1998**). "Specific interference by ingested dsRNA." *Nature*, 395(6705): 854.
